# Dabrafenib Alters MDSC Differentiation and Function by Activation of GCN2

**DOI:** 10.1158/2767-9764.CRC-23-0376

**Published:** 2024-03-13

**Authors:** M. Teresa Ciudad, Rene Quevedo, Sara Lamorte, Robbie Jin, Nadine Nzirorera, Marianne Koritzinsky, Tracy L. McGaha

**Affiliations:** 1 Tumor Immunotherapy Program, Princess Margaret Cancer Centre, University Health Network, Toronto, Canada.; 2 Department of Immunology, University of Toronto, Toronto, Canada.; 3 Princess Margaret Cancer Center, University Health Network, Toronto, Canada.; 4 Institute of Medical Science, University of Toronto, Toronto, Canada.; 5 Department of Radiation Oncology, University of Toronto, Toronto, Canada.; 6 Department of Medical Biophysics, University of Toronto, Toronto, Canada.

## Abstract

The effect of targeted therapeutics on anticancer immune responses is poorly understood. The BRAF inhibitor dabrafenib has been reported to activate the integrated stress response (ISR) kinase GCN2, and the therapeutic effect has been partially attributed to GCN2 activation. Because ISR signaling is a key component of myeloid-derived suppressor cell (MDSC) development and function, we measured the effect of dabrafenib on MDSC differentiation and suppressive activity. Our data showed that dabrafenib attenuated MDSC ability to suppress T-cell activity, which was associated with a GCN2-dependent block of the transition from monocytic progenitor to polymorphonuclear (PMN)-MDSCs and proliferative arrest resulting in PMN-MDSC loss. Transcriptional profiling revealed that dabrafenib-driven GCN2 activation altered metabolic features in MDSCs enhancing oxidative respiration, and attenuated transcriptional programs required for PMN development. Moreover, we observed a broad downregulation of transcriptional networks associated with PMN developmental pathways, and increased activity of transcriptional regulons driven by *Atf5*, *Mafg*, and *Zbtb7a*. This transcriptional program alteration underlies the basis for PMN-MDSC developmental arrest, skewing immature MDSC development toward monocytic lineage cells. *In vivo*, we observed a pronounced reduction in PMN-MDSCs in dabrafenib-treated tumor-bearing mice suggesting that dabrafenib impacts MDSC populations systemically and locally, in the tumor immune infiltrate. Thus, our data reveal transcriptional networks that govern MDSC developmental programs, and the impact of GCN2 stress signaling on the innate immune landscape in tumors, providing novel insight into potentially beneficial off-target effects of dabrafenib.

**Significance:**

An important, but poorly understood, aspect of targeted therapeutics for cancer is the effect on antitumor immune responses. This article shows that off-target effects of dabrafenib activating the kinase GCN2 impact MDSC development and function reducing PMN-MDSCs *in vitro* and *in vivo*. This has important implications for our understanding of how this BRAF inhibitor impacts tumor growth and provides novel therapeutic target and combination possibilities.

## Introduction

Myeloid-derived suppressor cells (MDSC) are immature monocytic and polymorphonuclear (PMN) lineage cells that are expanded and activated in response to malignancy (1). In cancer, MDSCs are defined by their potent ability to suppress T-cell activation and function, contributing to immune suppression and cancer progression. In the most widely accepted model of MDSC development, PMN and monocytic MDSCs (m-MDSC) are derived from hematopoietic progenitors that expand and acquire immune-suppressive function in response to tumor-driven emergency granulopoiesis. Supporting this developmental model, *in vitro* analysis has shown that addition of inflammatory cytokines promoted development of MDSCs with potent ability to suppress T-cell proliferation and effector function (2). However, increased insight regarding the impact of tissue microenvironmental cues, stress signaling, the inflammatory landscape (local and systemic), and epigenetics on MDSC development/function has highlighted the complexity of MDSC biology, which is clearly still poorly understood (3).

One important factor that influences MDSC physiology is intracellular and microenvironmental stress. For example, unfolded protein response (UPR) stress signaling transmitted by protein kinase R-like endoplasmic reticulum kinase (PERK) has a profound impact on MDSC suppressive function (4). Similarly, our group has reported that general control nonderepressible 2 (GCN2) is a key driver of MDSC function in the tumor microenvironment. Genetic deletion of *Eif2ak4* (the gene encoding GCN2) in myeloid cells altered MDSC metabolism and suppressive function enhancing antitumor CD8^+^ T-cell immunity *in vivo* (5). We also have found that GCN2 activation modulated macrophage and dendritic cell (DC) function in inflammatory disease altering cytokine production in endotoxemia (6) and driving acquisition of a suppressive phenotype in autoimmunity (7). The above findings suggested that relative GCN2 activity is an important modulating feature of myeloid biology regulating inflammatory and tolerogenic potential.

GCN2 is an evolutionarily ancient Ser/Thr kinase found in all eukaryotes that is an integral part of the integrated stress response (ISR), a cellular response system activated by diverse nutritional and environmental stress signals (8). GCN2’s kinase activity is induced by ribosomal stalling on mRNA transcripts resulting from paucity of aminoacyl-charged tRNA from amino acid starvation or active translation (9, 10). Once activated, GCN2 phosphorylates eukaryotic initiation factor (eIF)2α, significantly slowing GDP/GTP exchange in the translational complex abrogating cap-dependent translation (11). Slowed ribosomal assembly resulting from reduced GTP priming by eIF2α also induces a transcriptional stress response increasing translation of activating transcription factor 4 (ATF4) and ATF5 which drives expression of amino acid transporters and modulates metabolism, autophagy, and proliferation altering cellular phenotype and promoting survival (11).

It has become increasingly clear that chemotherapeutics, radiation, and targeted therapies have the potential to control cancer growth by off-target effects impacting the cancer cells themselves and/or the induction of anticancer immunity (12). In this vein, a growing body of literature suggests that target promiscuity by small-molecule kinase inhibitors impacting GCN2 activity contribute to their therapeutic efficacy. For example, neratinib, an EGFR inhibitor, activates GCN2 in glioblastoma cells contributing to its antitumor activity (13). Similarly, a kinome screen identified the EGFR inhibitor erlotinib and the receptor tyrosine kinase inhibitor sunitinib as agonists of GCN2 (13). Dabrafenib is a small-molecule kinase inhibitor used to treat BRAF-mutant (mut) cancers, including melanoma and non–small cell lung cancer (14, 15). Mass spectrometry‐-based chemical proteomics analysis of dabrafenib target profiles in melanoma cells identified a putative interaction with GCN2, a predicted property not shared by another BRAF inhibitor, vemurafenib (16). This interaction was confirmed in a chemical screen that identified dabrafenib as a potent GCN2 agonist (17).

Because we have previously shown that GCN2 is essential for MDSC phenotype impacting metabolism and immune function, in this study we examined whether dabrafenib-mediated modulation of GCN2 activity would impact MDSC development and function. Here, we show that direct activation of GCN2 by dabrafenib induced changes in MDSC differentiation blocking expansion and differentiation into PMN lineage cells. This altered development reshapes function by driving cells to acquire a phenotype with reduced suppressive capability.

## Materials and Methods

### Mice

C57BL/6 mice were purchased from the breeding colony at the Princess Margaret Cancer Centre animal facility. *C57BL/6-Tg(TcraTcrb)1100Mjb/J* (OT-I), *B6.PL-Thy1a/CyJ* (Thy1.1), and B6.129S6-Eif2ak4tm1.2Dron/J (GCN2^−/−^) mice were purchased from The Jackson Laboratory. All mice were housed under specific pathogen–free conditions in accordance with the Canadian Institutional Animal Care and Use Committee guidelines. Protocols were approved by the Princess Margaret Cancer Centre Animal Care Committee. Female mice 7–10 weeks of age were used for all experiments.

### Tumor Cell Culture and Tumor Injection

Mouse melanoma YUMM1.7 (RRID:CVCL_JK16) and YUMMER tumor cells (RRID:CVCL_C2VW) were obtained from ATCC and Sigma-Aldrich, respectively. Cells were cultured in DMEM/F-12 (Gibco), supplemented with 10% FBS, 100 units/mL of penicillin, 100 µg/mL of streptomycin, and 55 µmol/L 2-Mercaptoethanol. Cells were maintained at 37°C in 5% CO_2_-humidified atmosphere. A total of 3 × 10^5^ YUMM1.7 cells or 1 × 10^6^ YUMMER were injected subcutaneously into the right hind leg of each mouse. Cell lines were implanted at passage 6 to 10.

Screening for *Mycoplasma* and other rodent infectious agents in the cells lines was performed routinely using CLEAR PCR Panel at Charles River Laboratories, following the regulations by the Animal Resources Centre at University Health Network.

### BRAFi Formulation for *In Vivo* Treatments

Dabrafenib (30 mg/kg; Cayman Chemical) or the equivalent volume of DMSO were dosed orally once a day as a suspension in 0.5% Hydroxypropyl Methylcellulose (HPMC) and 0.2% Tween80 in distilled water at pH 8.

### Generation of Bone Marrow–derived MDSC

Bone marrow was flushed out of the tibia and femur. Red cells were depleted with Ammonium–Chloride–Potassium (ACK) lysing buffer. Cells were seeded at concentration 6 × 10^5^ cells/mL in RPMI1640 medium supplemented with 10% FBS, 100 units/mL of penicillin, 100 µg/mL of streptomycin, and 55 µmol/L 2-Mercaptoethanol. GMCSF (50 ng/mL) and IL6 (50 ng/mL) were used to drive MDSC differentiation. Cells were maintained at 37°C in 5% CO_2_-humidified atmosphere for 4 days. Alternatively, myeloid progenitors were isolated with EasySep Mouse Hematopoietic Progenitor Cell Isolation Kit (StemCell) and stimulated in the conditions mentioned above. Increasing concentrations of dabrafenib or matching DMSO volume were added at the time of seeding.

### Suppression Assays

Ovalbumin (OVA)-specific CD8^+^ T cells were isolated from the spleens and lymph nodes of OTI/Thy1.1 mice by negative selection (Stemcell Technologies) and labeled with carboxyfluorescein diacetate succinimidyl ester (CFSE) for proliferation tracking. CD11c^+^ cells were isolated from the spleens of C57BL/6 by positive selection (Stemcell Technologies) to serve as antigen-presenting cells. Briefly, CD11c^+^ cells were pulsed with 1 µg/mL OVA_257–264_ peptide for 1 hour at 37°C. After washing, cells were seeded at a ratio 1:10 CD11c^+^:CD8^+^ T cells in 96-well plates. MDSC were harvested and added to the cell mix at different ratios of MDSC:CD8^+^ T cells.

### Flow Cytometry and Cell Sorting

Single-cell suspensions of cells were stained in a staining buffer (PBS, 2% FBS, 1 mmol/L ethylenediaminetetraacetic acid [EDTA]) containing fixable viability Dye, Fc blocker, and the appropriate antibody cocktail. Samples were washed, fixed in 1% paraformaldehyde, washed again, and resuspended in staining buffer. Cells were acquired using a LSR Fortessa or LSR Fortessa X-20 analyzer (BD Biosciences) and data were analyzed using FlowJo software version 10.8.1 (Treestar). Alternatively, cells were FACSorted on FACSAria Fusion sorter (BD Biosciences) in tubes containing complete RPMI. See supplementary Table S1 for antibody details.

### RNA Isolation and qPCR

RNA from lysates was purified using RNeasy Plus RNA purification kit (Qiagen) and retrotranscribed using qScript cDNA supermix (Quanta bio). cDNA was amplified using the PerfeCTa SYBR green Supermix (Quanta bio) on a CFXConnect real-time PCR detection system (Bio-Rad). Results were analyzed using the accompanying software according to manufactures instructions. See Supplementary Table S2 for primer list.

### Western Blot Analysis

Fresh cells pellets were lysed in lysis buffer (1% Triton-X100, 50 mmol/L Tris-HCl, 100 mmol/L NaCl, pH 7.4) in presence of 1x protease and phosphatase inhibitors for 1 hour at 4°C while rotating. See Supplementary Table S3 for antibody details.

### Polysome Profiling

A total of 1 × 10^7^ cells per condition were used for polysome profiling as described previously (7), with some modifications. In brief, cells were treated with 0.1 mg/mL cycloheximide (CHX) for 3 minutes at 37°C and washed twice with ice-cold PBS/CHX. Cell pellets were then homogenized in 200 µL of lysis buffer [1% Triton X-100, 0.3 mol/L NaCl, 15 mmol/L MgCl_2_, 15 mmol/L Tris (pH 7.4), 0.1 mg/mL CHX, 100 units RNAse Inhibitors (Takara)] for 20 minutes at 4°C in a rotating mixer. The homogenate was centrifuged to remove nuclei, and supernatants were stored at −80°C until use. For each run, equal volumes of cell lysates were layered over a 10-mL linear sucrose gradient [20%–50% sucrose in 15 mmol/L, MgCl_2_, 15 mmol/L Tris (pH 7.4), 0.3 mol/L NaCl] and centrifuged for 90 minutes at 39,000 rpm in an SW41-Ti rotor at 4°C. Absorbance at 254 nm was measured continuously as a function of gradient depth in a Bio-Rad Laboratories UV monitor. Overall translation efficiency was calculated as area under the region of the curve that represented mRNA attached to two or more ribosomes compared with the total AUC.

### Seahorse Assay

*In vitro* differentiated MDSCs were seeded in Agilent Seahorse XF96 microplates coated with 15 µg/mL of Cell-Tak (Corning) at a density of 1 × 10^5^ cells per well. Extracellular acidification rate (ECAR) and oxygen consumption rate (OCR) were measured after injections of 25 mmol/L glucose, 1.5 µmol/L oligomycin A, 1.5 µmol/L FCCP, 1 mmol/L sodium pyruvate, 2.5 µmol/L antimycin A, and 1.25 µmol/L rotenone A optimizing a methodology described previously (18).

### Single-cell RNA sequencing and Analysis

MDSC were harvested and washed two times with PBS + 0.04% BSA. Then approximately 1 × 10^4^ cells were captured with 10X Genomics Chromium Next GEM Single Cell 5′ Kit v2. Libraries were subsequently prepared and sequenced using a NovaSeq sequencer (Illumina).

The single-cell RNA (scRNA) data were demultiplexed and converted to FASTQ file format with Illumina bcl2fastq. Initial quality control and alignment against mouse reference transcriptome Mm10-2020-A was performed on the resulting scRNA data using CellRanger (v7.0.0) count mode with SC5P-R2 chemistry. Briefly, Seurat (v4.3.0) was used to exclude any cells with >10% of the reads mapping to mitochondrial genes, cells that are considered as outliers (MAD>4) by expressing an excessive amount of genes/reads by scater (v1.22.0; ref. 19), and cells/clusters considered as doublets as identified by the intersection of DoubletFinder (v2.0.3; ref. 20) and scds (v1.9.1; ref. 21). We then performed normalization on the filtered count matrix and variance stabilization across 3,000 genes, regressing out cell-cycle and mitochondrial genes, using SCTransform v2 (22). Samples were integrated across treatment conditions using FastMNN as implemented in SeuratWrappers (v0.3.1) and defined from the batchelor (v1.10.0) R package (23). Principal component analysis (PCA) was performed, and the top 40 principal components were included in a Uniform Manifold Approximation and Projection (UMAP) for dimensionality reduction, as well as for finding shared nearest neighbors and the Leiden Clustering algorithm with a resolution of 0.9. Differentially expressed genes between treatment conditions were calculated per cluster and cell type using the Wilcoxon test implemented in the FindMarkers function of Seurat using no fold change or percent-expressed thresholds.

Cell type annotations were done by creating a reference atlas using 40 datasets that categorized features of developing and mature monocyte, PMN, and MDSC populations among others (Supplementary Table S4). Each dataset was preprocessed using methods like those outlined in this article, subSetted to have 1,000 cells representing each dataset and integrated together using FastMNN. Cell types were predicted per cell by estimating the shared nearest neighbor between the query and reference datasets using the function FindTransferAnchors of Seurat. These annotations were further refined by examining the differentially expressed genes across each cluster type.

Regulon analysis was performed using SCENIC (v1.2.4), pyScenic (v0.12.0), and their activity was scored using AUCell (v1.21.2; ref. 24). Genes were removed from this analysis if they were not expressed in at least 1% of all cells. Differential expression of regulons per cluster was performed using a Wilcoxon test between treatment conditions, corrected for multiple hypothesis testing using the Benjamini–Hochberg method, and regulons were deemed significant if they exceeded an |log_2_FC| > 0.01 and *q* < 0.01. Pathway analysis was performed on the MSigDB gene sets Hallmark, C2:Reactome, and C5:GOBP, accessed through the msigdbr (v7.4.1) R package. Significant differentially expressed pathways were identified using single-cell pathway analysis (SCPA) v1.5.2 (25) and their corresponding activity was estimated using AUCell.

Trajectory analysis was performed using monocle3 (v1.0.0; ref. 26) by importing the UMAP and PCA coordinates from the preconstructed Seurat object into the Single Cell Experiment object. Analysis was done according to the guide found at http://cole-trapnell-lab.github.io/monocle-release/monocle3. Selected branches were focused on by iterating through key start and end nodes, splitting the cells along that branch into treatment groups, and identifying variable genes along these paths with a *q*-value <0.05 and modularizing at a resolution of 0.001. To gauge the pathway and regulon activity across each branch, AUCell was used to score the activity of every cluster spanned by a given branch based on the average log-normalized expression of cells within that cluster and branch. Significant pathways and regulons were identified using an over-representation analysis as implemented in the clusterProfiler (v4.2.2) R package (27) of the gene modules on these same gene sets.

### Statistical Analysis

Statistical parameters calculated with GraphPad Prism v9.5 are described in each figure legend. Error bars indicate the SD. IC_50_ values were calculated by nonlinear regression. Student *t* tests or ANOVA were used for *P*-value calculations. Statistically significant differences (*P* < 0.05) are indicated by the exact *P* value in figures and legends.

### Data Availability Statement

Raw and processed single-cell RNA sequencing (scRNA-seq) data are available from Gene Expression Omnibus (GEO) under accession number GSE239496. Source code used to process the data and generate Figs. 5 and 6 can be found at https://github.com/mcgahalab/ly6gc_mice.

## Results

### Dabrafenib Impairs MDSC Proliferation and Differentiation

**FIGURE 1:**
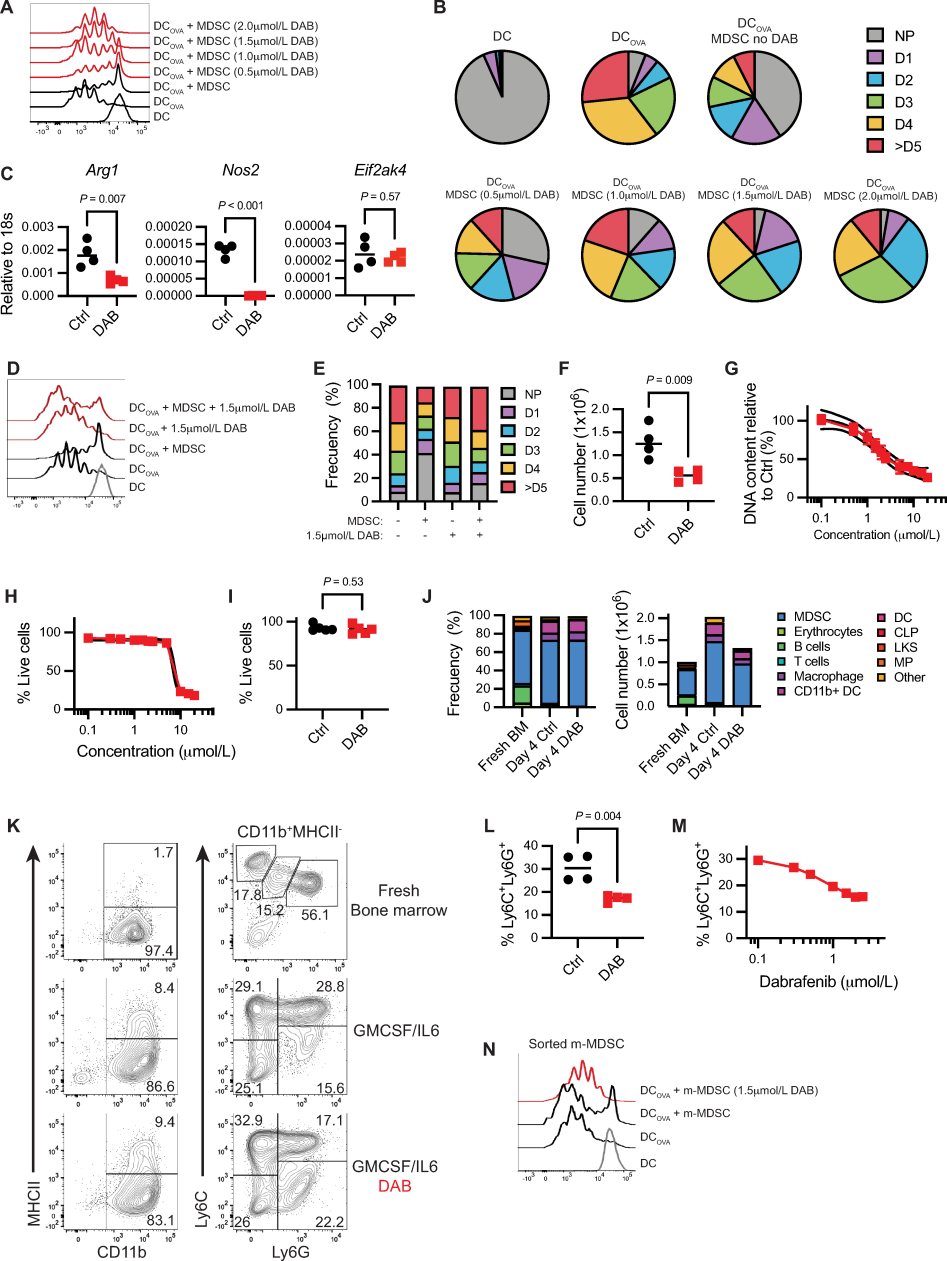
Dabrafenib (DAB) impairs MDSC proliferation and differentiation. **A,** Representative flow cytometry histograms showing antigen-specific proliferation of CD8^+^ T cells from OTI mice modulated by bone marrow–derived MDSC. T cells were cocultured with OVA_257–264_ peptide-pulsed DCs and MDSC at ratio DC: T cell: MDSC 1:10:30, where 1 = 10,000 cells, in 96-well plates for 60 hours. Experiment repeated three times with similar results. DAB = dabrafenib. **B,** Frequency of OTI CD8^+^ T undergoing one (D1), two (D2), three (D3), four (D4), or more than five (D5) cycles of division from plot A. NP = nonproliferative cells. Data are expressed as mean value. Experiment repeated three times with similar results. **C,** Mean *Arg1*, *Nos2, and Eif2ak4* expression relative to 18s RNA, assessed by qPCR (*n* = 4). MDSCs were generated in presence ± 1.5 µmol/L DAB for 4 days. *P* value was determined by two-tailed unpaired Student *t* test. Significance considered *P* < 0.05. Experiment repeated three times with similar results. **D,** Representative flow cytometry histograms showing antigen-specific proliferation of CD8^+^ T cells from OTI mice modulated by mature bone marrow–derived MDSC. T cells were cocultured with OVA_257–264_ peptide-pulsed DCs and MDSC at ratio DC: T cell: MDSC 1:10:30, where 1 = 10,000 cells, in 96-well plates for 60 hours in presence of ± 1.5 µmol/L DAB. Experiment repeated three times with similar results. DAB = dabrafenib. **E,** Frequency of OTI CD8^+^ T undergoing one (D1), two (D2), three (D3), four (D4), or more than five (D5) cycles of division from D. NP = nonproliferative cells. Data are expressed as mean value. Experiment repeated three times with similar results. **F,** Number of MDSCs generated *in vitro* in the presence of ± 1.5 µmol/L DAB after 4 days in culture (*n* = 4). *P* value was determined by two-tailed unpaired Student *t* test. Significance considered *P* < 0.05. Experiment repeated three times with similar results. **G,** Dose–response for inhibition of proliferation after 4 days in culture. DNA fluorescence was measured for cultures with the indicated concentration of dabrafenib. IC_50_ value was calculated at 1.59 µmol/L. Lines represent the mean ± SEM of four independent experiments. **H,** Dose–response for viability after 4 days in culture in presence of 1.5 µmol/L DAB. Each datapoint indicates the percentage of total live cells assessed by flow cytometry, normalized to control samples. Lines represent the mean ± SEM of four independent experiments. **I,** Percentage of live cells in MDSC *in vitro* cultures ± 1.5 µmol/L DAB assessed by flow cytometry. Error bars indicate ± SD (*n* = 4). *P* value was determined by two-tailed unpaired Student *t* test. Significance considered *P* < 0.05. Experiment repeated four times with similar results. **J,** Mean frequency and total cell number of different subsets of immune cells in fresh bone marrow (BM) and MDSC. A total of 1 × 10^6^ BM cells were seeded at the start of the experiment and differentiated for 4 days ± 1.5 µmol/L DAB (*n* = 4). Color coding represents each subtype. Experiment repeated two times with similar results. **K,** Representative flow cytometry contour plots of MDSC surface phenotype when expanded for 4 days with ± 1.5 µmol/L DAB compared with the ones found in fresh bone marrow. Gates show mean frequency (*n* = 4). Experiment repeated two times with similar results. **L** and **M,** Mean frequency of the PMN population CD11b^+^MHCII^neg^Ly6C^+^Ly6G^+^ in MDSC ± 1.5 µmol/L DAB (**J**) or increasing doses of DAB (K) (*n* = 4). Experiment repeated three times with similar results. In **J**, *P* value was determined by two-tailed unpaired Student *t* test. Significance considered *P* < 0.05. **N,** Representative flow cytometry histograms showing antigen-specific proliferation of CD8^+^ T cells from OTI mice modulated by FACS sorted m-MDSC generated in presence of ± 1.5 µmol/L DAB. T cells were cocultured with OVA_257–264_ peptide-pulsed DCs and MDSC at ratio DC: T cell: MDSC 1:10:20, where 1 = 5,000 cells, in 96-well plates for 60 hours. Experiment repeated three times with similar results. DAB = dabrafenib.

To study the impact of dabrafenib–GCN2 interaction on MDSC development, we utilized an *in vitro* model where bone marrow–derived cells are cultured in the presence of GMCSF and IL6 for 4 days to generate immune suppressive MDSCs (2, 5). First, we assessed the dabrafenib effect on MSDC suppressive function. Enriched splenic CD11c^+^ DCs were pulsed with ovalbumin peptide OVA_257–264_ and cocultured with transgenic T cells reactive against OVA_257–264_ [i.e., OTI CD8^+^ T cells (28)] in the presence or absence of MDSCs generated in a gradient of dabrafenib. Antigen-pulsed DCs drove robust T-cell proliferation (Fig. 1A and B), whereas addition of MDSCs reduced overall T-cell proliferation by 60%, increasing the fraction of undivided cells from 5% to 40% (Fig. 1A and B). Strikingly, MDSCs generated in a gradient of dabrafenib showed a dose-dependent decrease in the ability to suppress T-cell proliferation, indicating dabrafenib reduced MDSC suppressive activity. Increased enzymatic arginine metabolism is a hallmark feature of MDSC biology and suppressive function (29). Dabrafenib treatment significantly decreased expression of the arginine metabolizing enzymes such as arginase 1 (*Arg1*) and a complete abrogation of inducible nitric oxide synthase (*Nos2*), while it did not modulate GCN2 (*Eif2ak4*) gene expression (Fig. 1C). In addition, we tested the impact of dabrafenib in mature MDSC, where the drug was added during the T-cell suppression assay. While dabrafenib had no effect on T-cell proliferation after engaging DC-mediated stimulation, MDSC exposed to it lost their ability to suppress T-cell proliferation (Fig. 1D and E). These data show that dabrafenib had a profound impact on the MDSC immune-suppressive phenotype.

We then tested whether the reduced MDSC suppressive function in the presence of dabrafenib was the result of toxicity. We observed that the higher concentrations of dabrafenib reduced overall numbers of MDSCs (Fig. 1F). DNA content measurements indicated the IC_50_ for this effect was 1.59 µmol/L (SEM ± 0.1; Fig. 1G). To determine whether dabrafenib reduced cell numbers via toxicity, we quantified viability by flow cytometry. Cellular viability remained stable at approximately 89% (SEM ± 2.61) for dabrafenib concentrations below 10 µmol/L (Fig. 1H). Importantly, when using a dabrafenib concentration of 1.5 µmol/L (that reduced DNA content by 50%, Fig. 1G), viability was not compromised, suggesting the effect was not due to cell death (Fig. 1I).

We then asked whether dabrafenib impacted MDSC expansion or cellular composition (Fig. 1; Supplementary Fig. S1). In freshly isolated bone marrow, most cells expressed the general myeloid marker CD11b^+^ and a Ly6C^+^Ly6G^neg^ or Ly6C^+^Ly6G^+^ surface phenotype (57.8%, SEM ± 1.5). Ly6C^+^Ly6G^+^ cells represented the majority (56.1%, SD ± 2.9; Fig. 1J and K). In IL6+GMCSF expanded MDSC, CD11b^+^ cells were 94.3% (SD ± 1.8) of the total population after 4 days of culture (Fig. 1J and K). A total of 1.5 µmol/L dabrafenib did not have a significant effect on overall percentages of mature (CD11b^+^MHCII^+^) or immature (CD11b^+^MHCII^neg^) cells (Fig. 1J and K). However, PMN-MDSCs (Ly6C^+^Ly6G^+^) were significantly reduced from 28.8% (SD ± 5.1) to 17.1% (SD ± 4.5, *P* = 0.004; Fig. 1K and L). Dabrafenib-mediated reduction in the percentage of Ly6C^+^Ly6G^+^ cells was dose dependent (Fig. 1M), paralleling the decrease in DNA content observed (Fig. 1G) suggesting dabrafenib may impact PMN-MDSC differentiation in a selective fashion. In addition, when we FACS sorted the resulting m-MDSC, we observed that they were less immunosuppressive when generated in presence of dabrafenib (Fig. 1L).

### PMN-MDSCs Develop from Monocytes and Immature Progenitors

**FIGURE 2:**
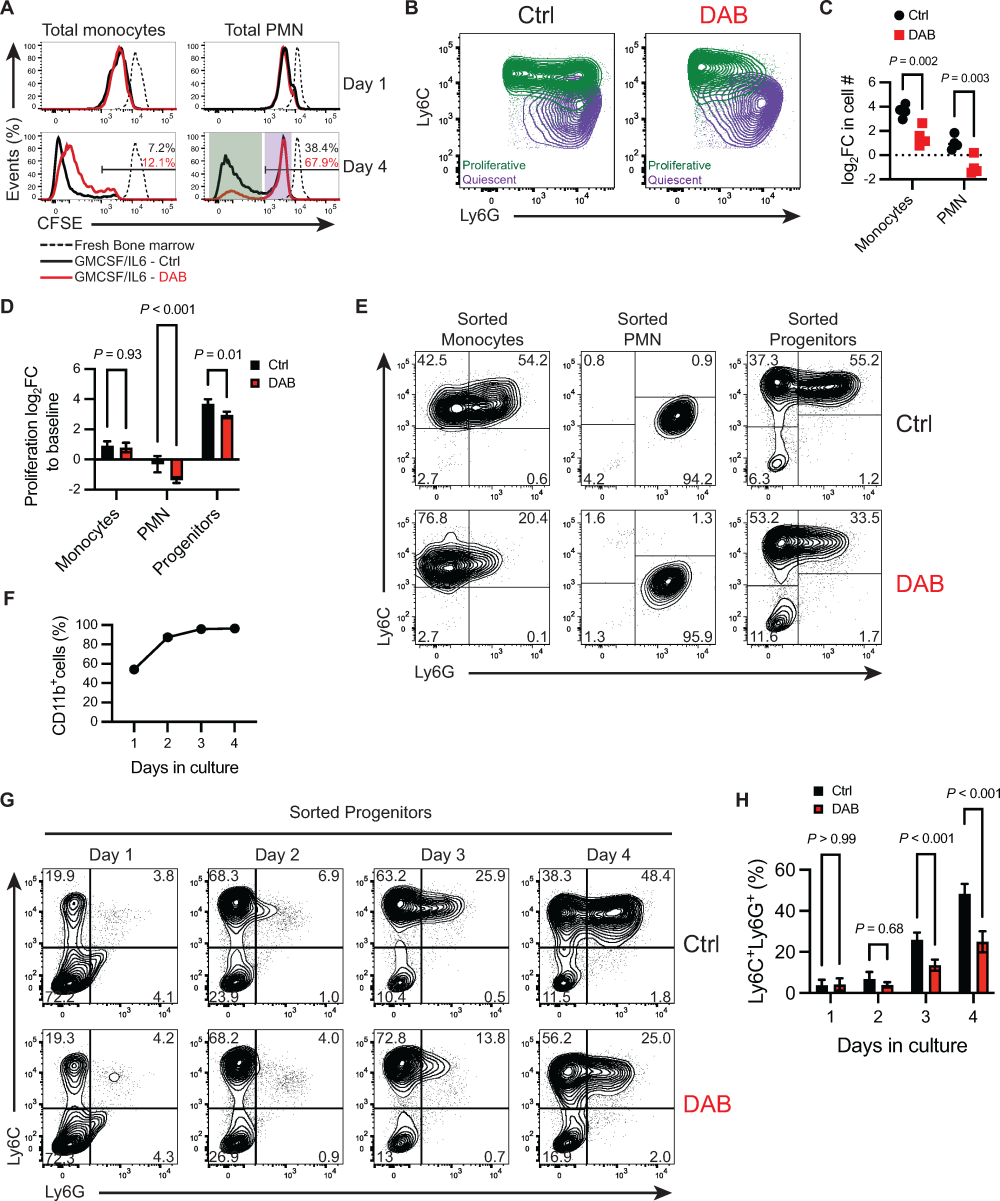
PMN-MDSCs develop from monocytes and immature progenitors. **A,** Representative flow cytometry histogram of CFSE dilution in total monocytes (CD11b^+^MHCII^neg^Ly6C^+^) and total PMN (CD11b^+^MHCII^neg^Ly6G^+^) on days 1 and 4 in culture ± 1.5 µmol/L DAB. Day 4 panels show percent (%) of CFSE^high^ cells. The purple shaded area shows PMN cells that remain quiescent and do not proliferate beyond the initial cycle on day 1. The green shaded area shows PMN cells that are highly proliferative. **B,** Representative flow cytometry contour plot of Ly6C and Ly6G expression in total PMNs gated as CD11b^+^MHCII^neg^Ly6G^+^ on day 4 after differentiation ± 1.5 µmol/L DAB. Quiescent (purple) and proliferative (green) PMN cells described in A are overlaid for each condition. **C,** log_2_ fold change (log_2_FC) of cell counts of total monocytes (CD11b^+^MHCII^neg^Ly6C^+^) and total PMNs (CD11b^+^MHCII^neg^Ly6G^+^) on day 4 normalized to the seeding counts of each lineage group at basal timepoint. MDSC were generated in presence of ± 1.5 µmol/L DAB. *P* values were determined by two-way ANOVA with Šidák correction post hoc-test. Significance considered *P* < 0.05. **D,** log_2_FC in proliferation measured by incorporation of DNA intercalating fluorescent dye on day 4, normalized to the fluorescence signal of cells at baseline. Sorted monocytes (CD3^neg^CD19^neg^CD11b^+^MHCII^neg^Ly6C^+^LyG6^neg^), PMN (CD3^neg^CD19^neg^CD11b^+^MHCII^neg^Ly6C^med^LyG6^+^) or progenitors (CD5^neg^CD11b^neg^CD19^neg^ B220^neg^Gr-1^neg^TER119^neg^) were differentiated to MDSC ± 1.5 µmol/L DAB. Cells were sorted from fresh bone marrow and plated at the same cell density (2 × 10^4^ cells/well). Bars show mean ± SD (*n* = 4). *P* values were determined by two-way ANOVA with Šidák correction post-test. Significance considered *P* < 0.05. **E,** Representative flow cytometry contour plot of Ly6C and Ly6G expression in CD11b^+^MHCII^neg^ cells differentiated for 4 days. Initial cell source was either sorted bone marrow monocytes (CD3^neg^CD19^neg^CD11b^+^MHCII^neg^Ly6C^+^LyG6^neg^), bone marrow PMNs (CD3^neg^CD19^neg^CD11b^+^MHCII^neg^Ly6C^med^LyG6^+^) or bone marrow progenitors (CD5^neg^CD11b^neg^CD19^neg^ B220^neg^Gr-1^neg^TER119^neg^). Gates show mean frequency. **F,** Frequency of total CD11b^+^ cells during sorted progenitor differentiation, assessed by flow cytometry for each day of cell differentiation. Line represents mean ± SD (*n* = 4). **G,** Representative contour plot of Ly6C and Ly6G expression in CD11b^+^MHCII^neg^ cells each day during sorted progenitor differentiation. Gates show mean frequency. Experiment repeated three times with similar results. **H,** Frequency of the granulocytic population CD11b^+^MHCII^neg^Ly6C^+^Ly6G^+^ during progenitor differentiation, determined by flow cytometry for each day of cell differentiation. Bars indicate mean ± SD (*n* = 4). *P* values were determined by two-way ANOVA with Šidák correction post-test. Significance considered *P* < 0.05. For all panels, experiments were repeated three times with similar results. DAB = dabrafenib.

On the basis of the above findings, we predicted that dabrafenib may impact expansion of PMN-MDSC precursors. To test this, we labeled total bone marrow with a proliferation tracer dye prior to the addition of IL6+GMCSF, monitoring dye dilution over a 4-day culture period. Dabrafenib did not impact early proliferation of either the monocytic (CD3^neg^CD19^neg^CD11b^+^MHCII^neg^Ly6C^+^) or PMN (CD3^neg^CD19^neg^CD11b^+^MHCII^neg^Ly6C^+^ Ly6G^+^) populations; however, proliferation was significantly inhibited at day 4 for both monocytic and PMN populations with the PMNs more severely impacted by dabrafenib (Fig. 2A). The monocytic population was actively proliferative with >90% of the cells exhibiting low amounts of tracer dye by day 4 (Fig. 2A); in contrast, PMN cells showed two distinct CSFE peaks on day 4. While the majority (61.6%, SD ± 6.1) showed a proliferative phenotype with low levels of tracer dye, 38.4% (SD ± 6.1) appeared quiescent without further cell division compared with day 1 (Fig. 2A). Dabrafenib treatment significantly expanded this quiescent PMN population at day 4 to 67.9% (SD ± 6.2) of the population while a minority of the PMN cells now appeared proliferative (32.1%, SD ± 6.2; Fig. 2A). The subset of PMN cells that were quiescent on day 4 exhibited a surface phenotype distinct from proliferative PMNs, with decreased Ly6C expression (Fig. 2B), likely forming the Ly6G^+^Ly6C^neg^ population observed when developing MDSCs were exposed to dabrafenib (Fig. 1I). However, analysis of absolute cell numbers showed that GMCSF+IL6 preferentially expanded the monocytic population (Ly6C^+^) 13.0-fold ±4.7 compared with fresh bone marrow (Fig. 2C). Despite being more numerous at culture initiation, Ly6G^+^Ly6C^+^ cells showed significantly less proliferative potential, expanding 2.2-fold ±1 relative to baseline numbers (Fig. 2C). Dabrafenib exposure significantly reduced Ly6C^+^ cell expansion, but some proliferative potential remained as we observed a 2.0-fold expansion in Ly6C^+^ cells compared with starting numbers (Fig. 2C). In contrast, Ly6G^+^ cells were severely impacted by dabrafenib and contracted by 50% at day 4 (Fig. 2C). These data suggest the reduction in cellularity by dabrafenib exposure was caused, at least partially, by arrested Ly6C^+^ cell expansion, and indicated that PMN-MDSCs (CD11b^+^MHCII^neg^Ly6C^+^Ly6G^+^) might have originated from monocytic lineage cells.

MDSCs exhibit plasticity in expansion and differentiation potential, promoting MDSC contribution to multiple mature myeloid populations in tissue microenvironments. Thus, to elucidate the cellular dynamics contributing to population development, we examined the effect of dabrafenib on expansion and differentiation of FACS-enriched myeloid precursors or more mature PMN and monocytic lineage cells. Sorted progenitors [lineage negative: CD5, CD11b, CD19, CD45R/B220, Ly6G/C(Gr-1), TER119, 7-4], monocytic (CD3^neg^CD19^neg^Ter119^neg^NK1.1^neg^CD11b^+^MHCII^neg^Ly6C^+^) or PMN (CD3^neg^CD19^neg^Ter119^neg^NK1.1^neg^CD11b^+^MHCII^neg^Ly6C^+^ Ly6G^+^) cells from bone marrow were seeded at the same cell density and cultured for 4 days with GMCSF+IL6 ±dabrafenib. Progenitors exhibited the greatest proliferative potential, expanding 6.9-fold more than enriched monocytic cells and 16.2-fold more than PMN cultures (Fig. 2D). Monocytic cells nearly doubled (log_2_FC = 0.9, SD ± 0.3), in stark contrast to isolated PMN populations that failed to expand in culture (Fig. 2D), indicating that the PMN accumulation observed in bulk cultures developed from progenitors and/or monocytic cells. Contrary to observations in bulk cultures, dabrafenib did not affect monocyte proliferation; however, dabrafenib accentuated PMN population contraction (Fig. 2C and D). Dabrafenib significantly reduced progenitor cell expansion (*P* < 0.01), suggesting that differential progenitor sensitivity to dabrafenib may be driving the observed effects on overall MDSC expansion (Fig. 2D). In progenitor cultures, exposure to IL6+GMCSF promoted development of monocytic Ly6C^+^Ly6G^neg^ and PMN Ly6C^neg^Ly6G^+^ MDSC populations (Fig. 2E). Similarly, monocytic cultures showed emergence of a Ly6C^+^Ly6G^+^ MDSC population after culture with GMCSF+IL6 for 4 days (Fig. 2E). Importantly, dabrafenib significantly reduced the Ly6C^+^Ly6G^+^ population for both monocytic and progenitor cultures (Fig. 2E). This contrasted Ly6C^neg^Ly6G^+^ PMN cultures which lacked the ability to differentiate into Ly6C^+^ MDSCs in the presence or absence of dabrafenib (Fig. 2E). Thus, the data suggest that dabrafenib limits differentiation plasticity of myeloid progenitors and newly generated m-MDSCs.

To better understand the impact of dabrafenib on MDSC development from progenitors, we monitored the kinetics of MDSC emergence from precursor populations. Total CD11b^+^ cells comprised 54% (SD ± 3) of all cells on day 2, and significantly increased up to 95.9% (SD ± 0.6) by day 3 (*P* < 0.001; Fig. 2F) an effect not impacted by dabrafenib. Monocytic Ly6C^+^Ly6G^neg^ MDSCs emerged as early as day 1 of culture, expanding to 68% (SD ± 4.3) of the total cultures by day 2. (Fig. 2G). In contrast, PMN-MDSCs did not significantly expand until culture day 3 where they were 25.9% (SD ± 3.6) of all MDSCs, further expanding to 48.4% (SD ± 4.7) on day 4 (Fig. 2G). Importantly, the Ly6G^+^ population emerged from the Ly6C^+^ myeloid population (Fig. 2G), consistent with the notion that PMN-MDSCs develop from the m-MDSCs. Dabrafenib did not impact m-MDSC expansion; however, PMN-MDSC expansion was significantly reduced (*P* < 0.001) on day 3 and the PMN-MDSCs that developed exhibited lower overall Ly6G expression (Fig. 2G and H). Thus, the data suggest inflammatory cytokines drive sequential evolution of MDSCs into Ly6C^+^ populations that lead to later emergence of PMN-MDSCs. Thus, transition from monocytic Ly6C^+^Ly6g^neg^ to PMN Ly6C^+^Ly6G^+^ MDSCs is attenuated by dabrafenib.

### Dabrafenib-induced GCN2 Activation Alters Proliferation and Differentiation of MDSCs

**FIGURE 3:**
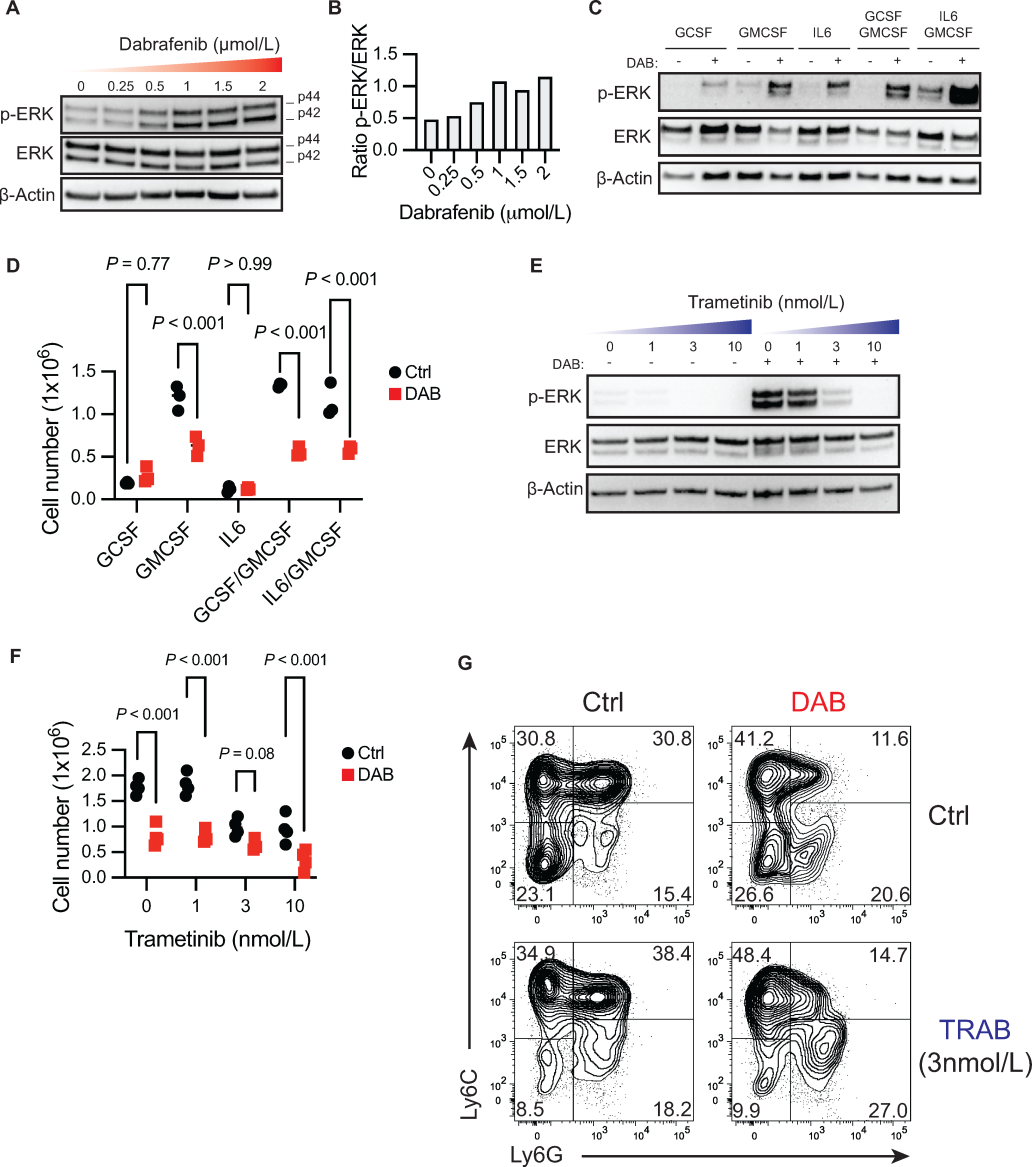
Activation of MAPK by dabrafenib is not responsible for phenotypic changes during differentiation. Western blot analysis (**A**) and relative quantification (**B**) of ERK1/2 phosphorylation (p-ERK) versus total protein at increasing doses of DAB in bone marrow–derived MDSC cultures on day 4 of differentiation with IL6+GMCSF. Western blot analysis of p-ERK (**C**) and total cell counts (**D**) of bone marrow cells differentiated with indicated cytokines ± 1.5 µmol/L DAB for 4 days. For **D**, *P* values were determined by two-way ANOVA with Šidák correction post-test. Significance considered *P* < 0.05. Western blot analysis of p-ERK (**E**) and total cell counts (**F**) of MDSC differentiated in presence of increasing doses of MEK inhibitor Trametinib (TRAB) ± 1.5 µmol/L DAB for 4 days. For F, *P* values were determined by two-way ANOVA with Šidák correction post-test. Significance considered *P* < 0.05. **G,** Representative contour plot of Ly6C and Ly6G expression in CD11b^+^MHCII^neg^ cells expanded in presence of 1.5 µmol/L DAB and/or 3 nmol/L TRAB. For all panels, experiments were repeated three times with similar results. DAB = dabrafenib.

Dabrafenib targets BRAF-mut; however, it also paradoxically hyperactivates the MAPK pathway in BRAF wild-type cells resulting in ERK1 and ERK2 activation (30). Accordingly, we observed a dabrafenib dose-dependent increase in phospho-(p)ERK1/2 in MDSCs with a 2-fold induction over baseline phosphorylation at 1 µmol/L (Fig. 3A and B). In control MDSCs, ERK1/2 activation was driven by a combination of IL6 and GMCSF signaling (Fig. 3C), an effect that was significantly enhanced by dabrafenib. Decreased cellularity due to dabrafenib was observed only when GMCSF was added to the cultures alone or in combination with other cytokines suggesting dabrafenib specifically impacts GMCSF-induced cellular expansion (Fig. 3D). Because dabrafenib enhanced ERK1/2 activation, we tested whether this was responsible for altered differentiation of MDSCs *in vitro*. For this, we used the MEK inhibitor trametinib to block ERK1/2 phosphorylation in dabrafenib-exposed MDSCs. A trametinib concentration of 3 nmol/L was sufficient to abrogate dabrafenib-induced p-ERK1/2 (Fig. 3E). However, MDSC cultures treated with trametinib had reduced cell numbers comparable to dabrafenib-treated MDSCs (Fig. 3F). Similarly, differentiation in presence of both dabrafenib and trametinib failed to restore dabrafenib-mediated PMN-MDSC phenotype (CD11b^+^MHCII^−^Ly6C^+^Ly6G^+^) observed in controls. (Fig. 3G). This shows that increased MAPK activity was not responsible for dabrafenib-mediated effects on MDSCs.

**FIGURE 4:**
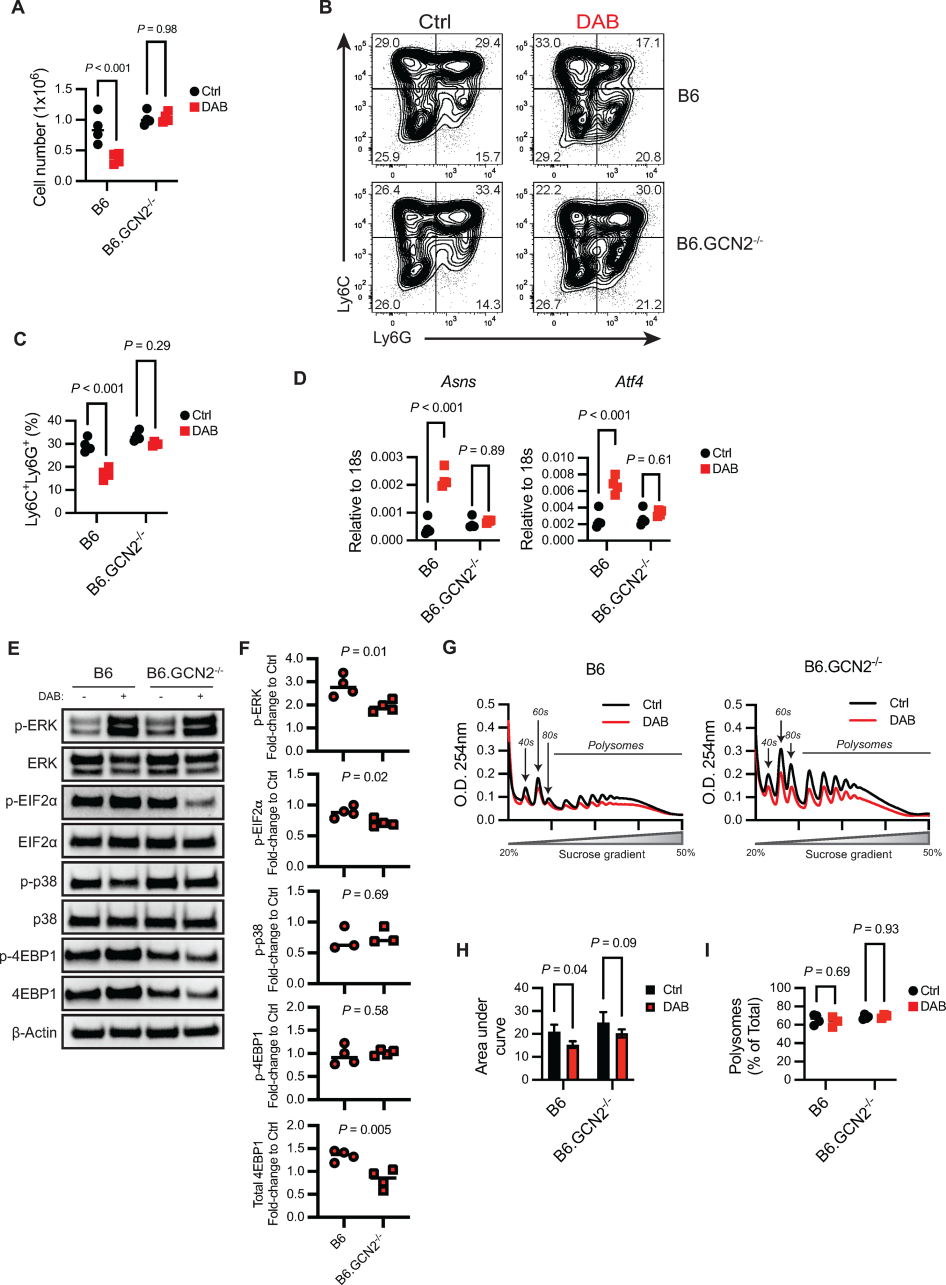
Dabrafenib-induced GCN2 activation alters proliferation and differentiation of MDSCs. **A,** Total cell counts in MDSC *in vitro* cultures from wild-type (B6) and GCN2-deficient (B6.GCN2^−/−^) mice differentiated in presence of ± 1.5 µmol/L DAB. *P* values were determined by two-way ANOVA with Šidák correction post-test. Significance considered *P* < 0.05. **B,** Representative flow cytometry contour plot of Ly6C and Ly6G expression in wild-type and GCN2^−/−^ MDSCs differentiated in presence of ± 1.5 µmol/L DAB. **C,** Frequency of CD11b^+^MHCII^neg^Ly6C^+^Ly6G^+^ MDSCs in wild-type and GCN2^−/−^ MDSCs differentiated in presence of ± 1.5 µmol/L DAB. *P* values were determined by two-way ANOVA with Šidák correction post-test. Significance considered *P* < 0.05. **D,** qPCR of *Asns* and *Atf4* in MDSCs lysates in wild-type and GCN2^−/−^ MDSCs differentiated in presence of ± 1.5 µmol/L DAB, relative to *18s* mRNA. *P* value was determined by two-tailed unpaired Student *t* test. Significance considered *P* < 0.05. Western blot analysis (**E)** and densitometry quantification (**F**) of ERK1/2, eIF2α, p38, and 4EBP1 phosphorylation relative to total protein in whole culture MDSC lysates. Quantification is shown as fold change in the intensity signal of samples treated with 1.5 µmol/L DAB compared with Ctrl sample for each genotype (wild-type or GCN2^−/−^). *P* value was determined by two-tailed unpaired Student *t* test. Significance considered *P* < 0.05. **G–I,** Ribosomal profiling analyzed in wild-type and GCN2^−/−^ MDSC lysates. **G,** Optical density (O.D.) at 254 nm. The area designated as “polysomes” represents the fraction of RNA-forming complexes of two or more ribosomes. **H,** AUC of the region marked as polysomes in G. Bars indicate mean ± SD (*n* = 4). *P* values were determined by two-way ANOVA with Šidák correction post-test. Significance considered *P* < 0.05. **I,** Relative quantification (%) of the AUC in polysomes versus monosomes (40s, 60s, and 80s region). Data are expressed as mean ± SD (*n* = 4). *P* values were determined by two-way ANOVA with Šidák correction post-test. Significance considered *P* < 0.05. For all panels, experiments were repeated three times with similar results. DAB = dabrafenib.

We then tested whether GCN2 activation was mechanistically required for dabrafenib modulation of MDSC differentiation. GCN2-deficient bone marrow was able to proliferate and develop into MDSCs in a manner comparable to GCN2 wild-type controls (Fig. 4A–C) suggesting GCN2 is not required for MDSC development. However, when GCN2 expression was lost, dabrafenib exposure no longer impacted expansion or differentiation of Ly6C^+^Ly6G^+^ MDSCs (Fig. 4A–C). Moreover, transcripts for the GCN2-responsive genes *Asns* and *Atf4* were significantly induced by dabrafenib, an effect that was completely abrogated in MDSC cultures lacking the gene coding GCN2, *Eif2ak4* (Fig. 4D). This shows GCN2 is activated by dabrafenib and GCN2 function is required for dabrafenib's MDSC inhibitory effects.

We next examined how dabrafenib–GCN2 interaction impacted ISR responses in MDSCs. eIF2α, the main target of GCN2 kinase activity, showed constitutive phosphorylation in control MDSC cultures, likely as a result of proliferative and translational stress (Fig. 4E and F). Loss of *Eif2ak4* did not impact basal p-eIF2α in MDSCs; however, addition of dabrafenib caused an unexpected 50% reduction in p-eIF2α in GCN2-deficient MDSCs compared with wild-type controls (Fig. 4E and F). Moreover, in GCN2^−/−^ MDSCs, there were reduced overall levels of 4EBP1, a negative regulator of translation (31) in treated conditions (Fig. 4E and F). In contrast, the dabrafenib-induced ISR response did not trigger cell death response though p38 activation (Fig. 4E and F). This suggested that in the absence of GCN2, dabrafenib reduced ISR signaling potentially enhancing translation. Thus, taken together, the data suggest GCN2 is activated by dabrafenib, and GCN2 stress signaling is the principal mechanism that drives altered MDSC differentiation.

We have shown previously that GCN2 activation can alter ribosome association with mRNA in myeloid cells resulting in a shift from polysomes to single ribosome-bound mRNA, a hallmark of reduced protein translation activity (7). Therefore, we examined whether dabrafenib treatment impacted RNA/ribosome association in MDSCs in a GCN2-dependent manner. Sucrose gradient analysis of RNA–ribosome complexes showed that dabrafenib reduced overall association of ribosomes with mRNA (Fig. 4G and H). However, it did not change the relative frequency of polysomes versus 40s/60s subunits or 80s ribosomes suggesting dabrafenib did not negatively impact polysome formation in a specific manner (Fig. 4I). Importantly, GCN2-deficient MDSCs exhibited a similar pattern of polysome association with mRNA (Fig. 4I). However, the dabrafenib-induced reduction in overall ribosome association with mRNA transcripts was attenuated in GCN2-deficient MDSCs (Fig. 4G and H). Thus, the data suggest that dabrafenib impacts development of MDSCs by a GCN2-dependent mechanism, with a general reduction in ribosome–mRNA interaction.

### Dabrafenib Induces Broad Transcriptional Changes in MDSCs

**FIGURE 5:**
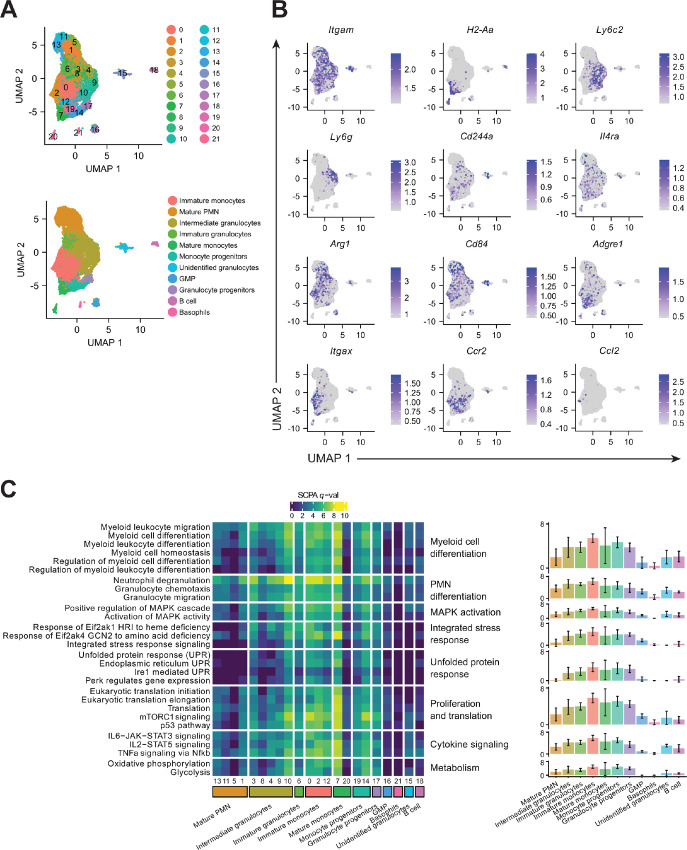
Dabrafenib induces broad transcriptional changes in MDSCs. **A,** Top, UMAP plot of the integrated scRNA-seq data from MDSC differentiated ± 1.5 µmol/L DAB, showing the 22 clusters defined by Seurat analysis. Bottom, UMAP plot of the integrated scRNA showing the 11 annotated lineage clusters. **B,** UMAP plot of the integrated scRNA-seq data from MDSCs showing RNA expression of selected myeloid markers. Scale represents normalized RNA expression. **C,** Heat map of the SCPA multivariate distribution analysis of genes associated with the Hallmark, Reactome, and Gene Ontology (GO) Biological process pathways. Pathways are representative top biological processes involved in myeloid cell and PMN differentiation, MAPK activation, ISR, UPR, proliferation and translation, cytokine signaling, and respiration metabolism. Left panel shows *q*-value for individual pathways in each cluster. Right panel shows the average *q*-value ±SD for the pathways included in the biological process indicated, for each annotated group. Higher *q*-values represent pathways with greater differences upon stimulation with DAB versus control. *q*-value is defined by SCPA as the square root of the log_10_(*P*-adjusted values) based on the Rosenbaum cross-match test.

GCN2-induced transcriptional responses are key drivers of its effect on myeloid cell phenotype (5); therefore, we next analyzed the transcriptomic responses in dabrafenib-treated MDSC subpopulations by scRNA-seq. Most cells were identified as myeloid, expressing *Itgam* (the gene encoding CD11b), with varying expression of MHC molecules (e.g., *H2-Aa*) and lineage identity markers (e.g., *Ly6c2* and *Ly6g*; Fig. 5A and B). To facilitate the proper annotation of the clusters, we integrated and annotated 40 datasets from publicly available data in GEO database spanning bone marrow enriched subpopulations, including different sets of precursors, as well as monocytic and PMN cells isolated from bone marrow and peripheral tissues in healthy and disease conditions (Supplementary Fig. S2A). This reference atlas was used to project our MDSC scRNA-seq onto. Annotation prediction (Supplementary Fig. S2B) as well as differential expressed genes (Supplementary Fig. S3) were used to curate the assignations. Cluster identity analysis revealed an array of bone marrow enriched developmental cell types including immature monocyte and PMN precursors and more mature monocytic and PMN populations. Four clusters were transcriptionally similar to mature PMNs found in the peritoneal cavity (clusters 1, 5, 11, and 13) expressing *Cfs3r*, *Mmp9*, *Cd9*, and *Il1rn* (ref. 32; Supplementary Fig. S3). In addition, five clusters (clusters 3, 4, 8, 9, and 10) were transcriptionally similar to developing PMNs found in the bone marrow, suggesting a more intermediate phenotype (33), while cluster 6 was transcriptionally similar to immature, Ly6G^lo^ neutrophils found in the peritoneal cavity (32) characterized by expression of several immunomodulatory chemokines (Supplementary Fig. S3). Three clusters (clusters 0, 2, and 12) identified as immature monocyte lineage cells characterized by expression of *Spp1*, *Lpl*, *Mgl2*, *Mmp12*, and *Ccr2* (Fig. 5B; Supplementary Fig. S3). There were two clusters (cluster 7, 20) with a mature monocyte transcriptional profile including expression of genes related to antigen presentation, migration, and recruitment (Supplementary Fig. S3).

To evaluate the overall impact of dabrafenib in each cluster, we performed multivariate distribution analysis with SCPA for each cluster comparing dabrafenib with controls (25). As expected, processes involved in myeloid and granulocyte differentiation were among the top regulated pathways in most clusters (adjusted *P*-value <0.01) as shown by a higher *q*-value statistic (Fig. 5C). Importantly, higher *q*-values were observed in immature granulocyte and immature monocyte clusters and, to a lesser extent, precursor clusters supporting the prediction that dabrafenib modulates differentiation at stages where cells still possess plasticity. Moreover, MAPK pathway regulation and GCN2 response genes were also significantly influenced by dabrafenib while the UPR was mildly impacted (Fig. 5C). In addition, dabrafenib treatment modulated proliferation and translation as well as STAT3, STAT5, and NFκB transcriptional modules in most clusters (Fig. 5C).

### MDSC Differentiation Arrest is a Result of a Combination of Cell Cycle and Myeloid Lineage Alteration

**FIGURE 6:**
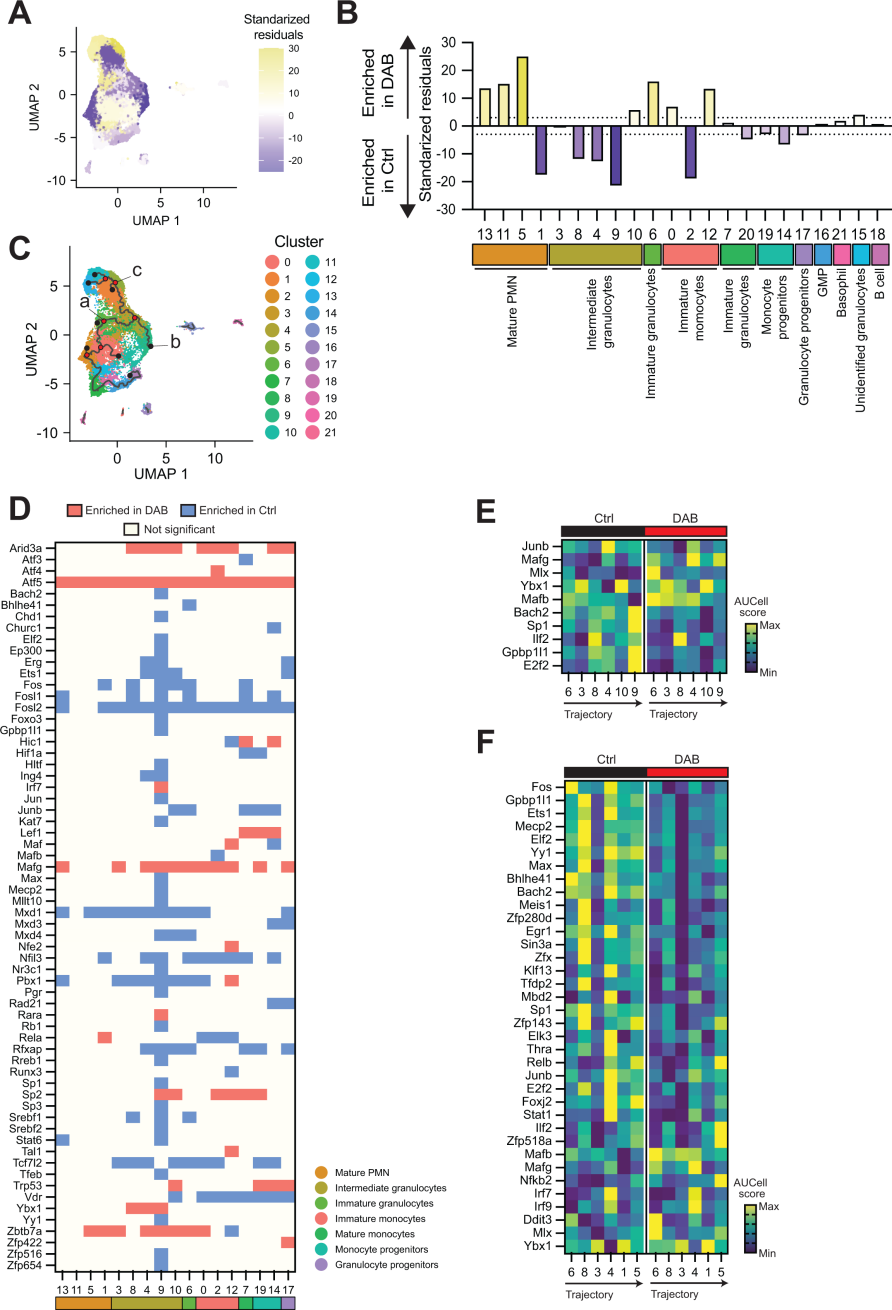
MDSC differentiation arrest is a result of a combination of cell cycle and myeloid lineage alteration. **A** and **B,** Relative frequency of cells in each Seurat cluster from scRNA-seq data of MDSCs ± 1.5 µmol/L DAB. Relative levels were calculated on the basis of the standardized residuals from using Pearson *χ*^2^ overlaid on the UMAP plot of integrated samples (**A**) and grouped by functional annotation (**B**). Positive standardized residuals (yellow) for each cluster indicates increased frequency in the DAB-treated sample whereas a negative number (purple) indicates increased frequency in the DMSO control sample. A dotted line indicates standardized residual values of ±3. **C,** UMAP plot of the integrated scRNA-seq data from MDSCs ± 1.5 µmol/L DAB, showing the trajectory branches calculated by Monocle3 and represented over the 22 clusters defined by Seurat. Most relevant nodes at the end of the branches (black dots) and at intersections (red dots) are shown. Nodes used as starting (a) or end (b and c) points for regulon analysis across the branch are labeled. **D,** Heat map of the regulons significantly regulated by DAB calculated on AUCell (*q* < 0.01, |log_2_FC| >0.01) for each Seurat cluster. Color represents regulons significantly enriched in cells treated with DAB (red), those enriched in control samples (blue) and the ones that do not show significant changes (light yellow). **E,** Heat map of the regulons significantly regulated during differentiation in the direction from node a to node b in C, which spans from cluster 6 (immature granulocytes) to cluster 9 (intermediate granulocytes) in DMSO control cell cultures. Scale represents z-score of the normalized average AUCell score per regulon. **F,** Heat map of the regulons significantly regulated during differentiation in the direction from node a to node c in C, which spans from cluster 6 (immature granulocytes) to cluster 5 (mature PMN) in DMSO control cell cultures. Scale represents z-score of the normalized average AUCell score per regulon. DAB = dabrafenib.

We identified the stage at which dabrafenib induced developmental arrest by evaluating the variation in cell frequency for each cluster. The relative frequency of cells in dabrafenib versus DMSO samples was calculated per each individual cluster using Pearson *χ*^2^ test where standardized residuals indicated deviation away from the null assumption of equal proportions and values greater than ±3 indicated a substantial difference (Fig. 6A). Using this approach, we identified three intermediate clusters (cluster 4, 8, 9) and one mature PMN cluster (cluster 1) significantly reduced by dabrafenib (Fig. 6B). Interestingly, the second most affected cluster was classified as immature monocytes (cluster 2) suggesting that monocyte subset differentiation is also compromised by dabrafenib (Fig. 6B). Likewise, dabrafenib exposure was also associated with accumulation of mature PMN clusters 5, 11, 13 (Fig. 6B). This indicates that dabrafenib primarily impacts the maturation state of PMN-MDSCs, reducing developmental intermediate populations in favor of more mature neutrophil populations.

In contrast, PMN cluster 6 and monocytic cluster 12 were the only immature populations enriched by dabrafenib treatment (Fig. 6B), suggesting they may represent transitional maturation stages where development is arrested influencing overall changes in cellular composition. To test this hypothesis, we performed trajectory analysis in the control DMSO sample which confirmed that cluster 6 is an early-stage founding node for PMN lineage differentiation (Fig. 6C). One branch followed the path toward more differentiated PMN stages while cluster 4 appeared to be a divergent node for an intermediate phenotype (cluster 9) or more mature stages (cluster 1, 5, 11, 13; Fig. 6C). The other branch connected to monocytic clusters and continued to progenitor clusters reinforcing the concept that such granulocytes derived from monocytic lineage cells. Taken together with the flow cytometry data, trajectory analysis suggests dabrafenib impacted MDSC differentiation at two stages: (i) dabrafenib impedes the transition from monocyte-to-PMN in cluster 12; and (ii) dabrafenib blocks early PMN differentiation from cluster 6 to more mature differentiation states.

To test this prediction, we performed downstream regulon analysis using the SCENIC package and calculated the AUCell enrichment scores for comparison between the treatment groups, focusing on the main group of clusters that showed interconnected trajectory branches. Like pathway analysis, we observed a generalized regulation of predicted transcription factor activity (*q* < 0.01, |log_2_FC| >0.01). Dabrafenib induced enrichment of *Atf5*, *Mafg,* and *Zbtb7a* regulons in the majority cell clusters (Fig. 6D; Supplementary Fig. S4A). *Atf5* is directly regulated by GCN2 activation (34), and its enrichment in all clusters supports the prediction of broad, dabrafenib mediated, GCN2 induction. *Zbtb7a* regulates hematopoietic development and glycolysis, thus its activation is likely to have a significant impact on cellular metabolism and development (35, 36). Likewise, the Maf transcription factor family impacts myeloid cellular identity and maturation (37), and *MAFG* expression is most abundant in intermediate neutrophil precursors with significant downregulation in mature PMNs as they exit the bone marrow (38). *Mxd1*, *Tcf7l2,* and *Fosl2* regulons were enriched in control samples suggesting dabrafenib inhibited their activity. Importantly, decreased enrichment of the *Mxd1* regulon was found in PMN clusters preferentially in dabrafenib treatment conditions (Fig. 6D; Supplementary Fig. S4A). *Mdx1* codes for MAD1, a negative regulator of MYC and downstream ribosome biogenesis (39). Moreover, MAD1 binds DNA complexes during PMN differentiation (40, 41) and influences fitness and maturation of conventional DCs (42). Thus, a loss of the *Mdx1* regulon may be associated with reduced ribosomal fitness and differentiation potential, which would be in line with our observations.

Focusing on cluster 12 (immature myeloid), which represented a key point of cell accumulation, we identified four regulons that were exclusively regulated by dabrafenib in this monocytic cluster. Cluster 12 showed enriched *Nfe2* and *Tal1* regulons after dabrafenib treatment with decreasing *Runx3* regulon activity (Fig. 6D; Supplementary Fig. s4B). TAL1 is a cell cycle activator in myeloid cells that inhibits expression of p21 and p16 (43) while RUNX3 is required for development and anti-inflammatory functions of mononuclear cells (44) providing a transcriptional basis for increased accumulation of cluster 12 (Fig. 6B) and reduced overall T-cell suppressive activity after dabrafenib exposure (Fig. 1A). In addition, we observed that PMN cluster 9 exhibited the highest number of downregulated transcriptional circuits with reduced enrichment of 36/44 regulons (Fig. 6D), paralleling the significant decrease in cluster abundance after dabrafenib treatment (Fig. 6B) suggesting these regulons are key drivers of PMN-MDSC development and function.

To better understand the influence of transcription factors programs on MDSC development ± dabrafenib, we calculated regulon enrichment in the trajectory branches identified. Because loss of PMN-MDSCs was the main effect of dabrafenib exposure, we focused on the importance of cluster 6 as the nascent population for developmentally intermediate PMN (cluster 9) and mature PMN-MDSCs (cluster 5). The trajectory analysis through cluster 6 to cluster 9 revealed *Bach2*, *Gpbp1l1,* and *E2f2* are regulons that are progressively activated in the developmental trajectory to intermediate PMNs (Fig. 6E). Those regulons were among the 22 regulons specifically modulated by dabrafenib in cluster 9 (Fig. 6D) suggesting their activation was necessary to differentiate to an intermediate PMN phenotype. *Rb1* is a regulator of PMN differentiation in a recently characterized monocytic progenitor subtype of committed neutrophil precursors (45). We identified *Rb1* regulon as significantly modulated by dabrafenib in cluster 9 (Fig. 6D) but was not identified in the cluster 6 to cluster 9 trajectory branch (Supplementary Fig. S4C) suggesting that differential retinoblastoma (Rb) was not driving PMN maturation in our samples.

We identified 36 regulons that were differentially regulated along the cluster 6 to cluster 5 trajectory branch in control cultures (Fig. 6F). Dynamic upregulation of their activity occurred during maturation progression and dabrafenib showed a broad inhibitory effect on regulon activity in general (Fig. 6F). In contrast, the regulon controlled by MAFG was increased by dabrafenib in this differentiation branch. The *Mafg* regulon showed relatively low enrichment in intermediate PMN clusters in controls; however, in dabrafenib-treated cells, the *Mafg* regulon was significantly enriched (Fig. 6E and F). This suggests precise regulation of *Mafg* is essential for control of PMN-MDSC.

**FIGURE 7:**
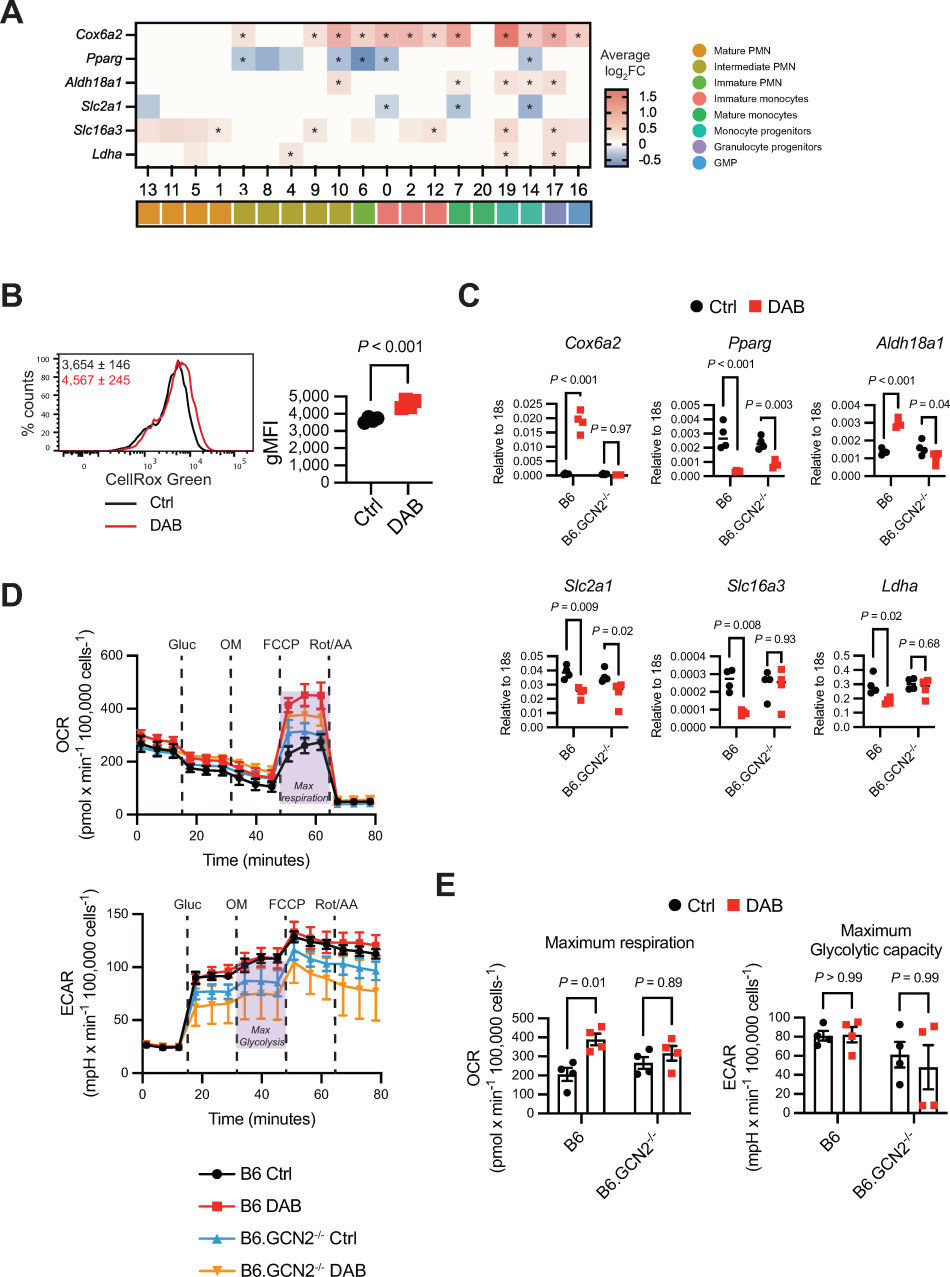
Dabrafenib increases oxidative metabolism in MDSCs. **A,** Heat map of the average log_2_FC in gene expression for the genes indicated ± 1.5 µmol/L DAB clusters identified in MDSC scRNA sequencing data described in Fig 6. Positive values indicate genes with induced expression by DAB, while negative values show genes that are downregulated by DAB. For representation, log_2_FC = 0 indicates clusters that were excluded from the analysis with *FindAllMarkers* function (gene expressed in <10% cells and/or log_2_FC ±0.25). Statistics show Wilcoxon test. * = adjusted *P*-value <0.05. **B,** Representative flow cytometry histogram of CellROX Green (ROS in mitochondria and nucleus) in wild-type MDSC generated with 1.5 µmol/L DAB (red line) or control (black line). Panels show geometric mean fluorescence intensity (gMFI) ±SD (*n* = 4). *P* value was determined by two-tailed unpaired Student *t* test. Significance considered *P* < 0.05. Experiment was repeated three times with similar results. **C,** qPCR of genes related to oxidative respiration (*Cox6a2*), fatty acid metabolism (*Pparg*), and glycolysis (*Aldh18a1, Slc2a1, Slc16a3, Ldha*) in total wild-type and GCN2^−/−^ MDSC lysates, normalized to *18s* mRNA expression(*n* = 4). *P* values were determined by two-way ANOVA with Šidák correction post-test. Significance considered *P* < 0.05. Experiment was repeated three times with similar results. **D,** Quantification of oxidative respiration by OCR and glycolysis by ECAR, by Seahorse assay. Lines represent the mean ±SEM of four independent experiments. Gluc = Glucose; OM = Oligomycin; FCCP = Carbonyl cyanide-p-trifluoromethoxyphenylhydrazone; Rot/AA = Rotenone/Antimycin. **E,** Maximum respiration calculated from OCR data and maximum glycolytic capacity calculated from ECAR data. Bars represent mean ± SEM of four independent experiments. *P* values were determined by two-way ANOVA with Šidák correction post-test. Significance considered *P* < 0.05. DAB = dabrafenib.

We have shown that GCN2 activity can alter MDSC metabolism, impacting MDSC function. Because several of the regulons identified can modulate metabolism and cellular energetics, we asked whether dabrafenib alters MDSC metabolism by GCN2 activation. First, we examined expression of metabolism-associated genes in the MDSC single-cell sequencing dataset, using a gene set we had previously identified as differentially expressed GCN2-deficient tumor macrophages and MDSC (5). *Cox6a2* (encoding a subunit of cytochrome C oxidase), was strongly induced by dabrafenib in monocytic and progenitor clusters; however, there was no apparent induction in the more mature PMN clusters except for the key intermediate PMN cluster 9 (Fig. 7A). Expression of genes involved in mitochondrial respiration (*Pparγ*), or glycolysis (*Aldh18a1*, *Slc2a1*, *Slc16a3*, *Ldha*) were also impacted by dabrafenib; however, the effect was less penetrant overall suggesting dabrafenib may enhance mitochondrial respiration (Fig. 7A). Oxidative respiration is one of the major endogenous sources of reactive oxygen species (ROS) and can induce ribosome stalling and GCN2 activation. We measured ROS by staining MDSCs with CellROX for 1 hour before analysis. We observed that dabrafenib increased mitochondrial and nuclear ROS (Fig. 7B). This suggested that dabrafenib increased mitochondrial respiration in MDSCs. Supporting this, mRNA measurements in bulk dabrafenib-treated MDSC cultures also demonstrated strong induction of *Cox6a2*, an effect that was completely abrogated by loss of GCN2 function (Fig. 7C). We then measured metabolic flux by seahorse, which showed that dabrafenib increased maximum oxidative respiration in the cultures 2-fold compared with controls but did not affect glycolytic flux (Fig. 7D and E). Moreover, in agreement with the mRNA analysis, loss of GCN2 function prevented dabrafenib-induced oxidative capacity increase (Fig. 7D and E). GCN2-deficient MDSCs showed a slight decrease in glycolysis overall, but glycolytic metabolic function was not impacted by dabrafenib (Fig. 7D and E). Thus, the data show that dabrafenib impacts metabolism, increasing oxidative respiration by activation of GCN2.

### Dabrafenib Reduced MDSC Accumulation in the Tumor Microenvironment

**FIGURE 8:**
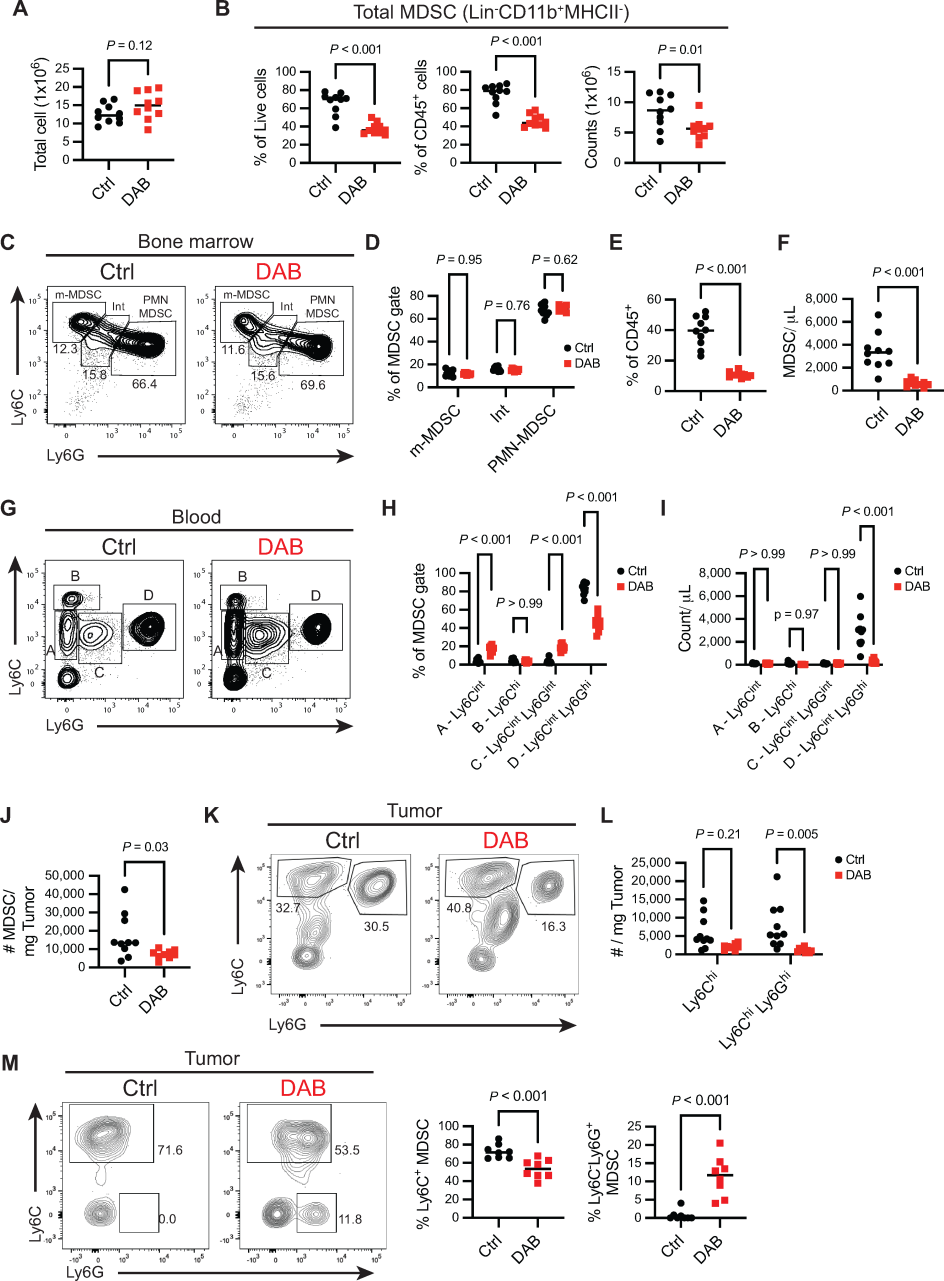
Dabrafenib reduces MDSC accumulation in the tumor microenvironment. Mice were implanted with 3 × 10^5^ YUMM1.7 cells subcutaneously in the right flank. On day 10 after tumor implantation, mice were either treated with 30 mg/kg of dabrafenib or the equivalent volume of DMSO for 7 consecutive days (*n* = 10 per group). Mice were sacrificed on day 20 after tumor implantation. **A,** Number of total live cells in bone marrow, isolated from one tibia and femur per mouse after red cells lysis. *P* value was determined by two-tailed unpaired Student *t* test. Significance considered *P* < 0.05. **B,** Frequency of MDSCs (CD45^+^CD3^neg^CD19^neg^CD11b^+^MHCII^neg^) from total live cells (left), from immune cells (middle), and absolute counts of MDSCs (right) in bone marrow. *P* value was determined by two-tailed unpaired Student *t* test. Significance considered *P* < 0.05. **C,** Representative contour plot of Ly6C and Ly6G expression in total MDSCs (CD45^+^CD3^neg^CD19^neg^CD11b^+^MHCII^neg^) in bone marrow. Gates show median frequency of m-MDSCs (Ly6C^+^Ly6G^neg^), PMN-MDSCs (Ly6C^+^Ly6G^+^) and cells with intermediate phenotype (Int, Ly6C^int^Ly6G^int^). **D,** Frequency of MDSCs subtypes described in C. *P* values were determined by two-way ANOVA with Šidák correction post-test. Significance considered *P* < 0.05. **E,** Frequency of MDSCs (CD45^+^CD3^neg^CD19^neg^CD11b^+^MHCII^neg^) from total immune cells. *P* value was determined by two-tailed unpaired Student *t* test. Significance considered *P* < 0.05. **F,** Number of circulating MDSCs (CD45^+^CD3^neg^CD19^neg^CD11b^+^MHCII^neg^) per microliter of blood. **G,** Representative contour plot of Ly6C and Ly6G expression in total MDSCs (CD45^+^CD3^neg^CD19^neg^CD11b^+^MHCII^neg^) in blood. Gates show simplified nomenclature for all four subpopulations Ly6C^int^ (A), Ly6C^hi^ (B), Ly6C^int^Ly6G^int^ (C), and Ly6C^int^Ly6G^hi^ (D). **H,** Frequency of MDSC subtypes described in F. *P* values were determined by two-way ANOVA with Šidák correction post-test. Significance considered *P* < 0.05. **I,** Absolute counts of MDSC subtypes described in F and G per microliter of blood. *P* values were determined by two-way ANOVA with Šidák correction post-test. Significance considered *P* < 0.05. **J,** Number of intratumoral MDSCs (CD45^+^CD11b^+^MHCII^neg^) per milligram of tumor. *P* value was determined by two-tailed unpaired Student *t* test. Significance considered *P* < 0.05. **K,** Representative contour plot of Ly6C and Ly6G expression in total intratumoral MDSCs (CD45^+^CD3^neg^CD19^neg^NK1.1^neg^CD11c^neg^CD11b^+^MHCII^neg^). Gates show median frequency of m-MDSCs (Ly6C^hi^) and PMN-MDSCs (Ly6C^hi^Ly6G^hi^). **L,** Absolute counts of MDSC subtypes described in J per milligram of tumor. *P* values were determined by two-way ANOVA with Šidák correction post-test. Significance considered *P* < 0.05. **M,** Mice were implanted with 1 × 10^6^ YUMMER cells subcutaneously in the right flank and 12 days later treated with dabrafenib as described above. Plots are representative contour plots of Ly6C and Ly6G expression in total intratumoral MDSCs (CD45^+^CD3^neg^CD19^neg^NK1.1^neg^CD11c^neg^CD11b^+^MHCII^neg^). Graphs show relative frequency of m-MDSCs (i.e., Ly6C^hi^, left graph) and more mature Ly6G^+^Ly6C^neg^ PMN cells from total MDSCs (right graph). *P* value was determined by two-tailed unpaired Student *t* test. Significance considered *P* < 0.05. All panels are representative of three independent experiments. DAB = dabrafenib.

We then tested whether dabrafenib would impact MDSC accumulation *in vivo* in the YUMM1.7 model of melanoma. YUMM1.7 possesses several of the hallmark driver mutations in melanoma including loss of *Pten* and *Cdkn2a* and the dabrafenib sensitive Braf^V600E^ mutation (46). YUMM1.7 cells were implanted in the right hind leg and on day 10, mice were allocated into the treatment groups according to their tumor volume to reduce tumor size variability between groups. Mice were then treated for 7 days with 30 mg/kg dabrafenib and analyzed by flow cytometry for MDSC composition in the bone marrow, blood, and tumor. In the bone marrow, dabrafenib treatment did not alter the number of live cells (Fig. 8A); however, MDSC numbers were reduced by 50% compared with control mice (Fig. 8B). In agreement with the *in vitro* data, 70% of the CD11b^+^MHCII^neg^ cells in the bone marrow were Ly6C^int^Ly6G^+^ PMN lineage cells (Fig. 8C and D). Although there was no reduction in m-MDSC versus PMN-MDSC populations after dabrafenib treatment [ratio PMN/m-MDSC 5.5 ± 1.7 in Ctrl and 6.0 ± 0.6 in dabrafenib (DAB); Fig. 8D], the dominance of PMN lineages in the MDSC gate (66.4% ± 5.1% and 69.6% ± 2.5% in Ctrl and DAB, respectively) suggests these cells are impacted by dabrafenib in agreement with our *in vitro* observations.

MDSCs (CD45^+^CD3^neg^CD19^neg^CD11b^+^MHCII^neg^) were 40.7% (SD ± 11.1) of the total CD45^+^ cells in circulation (Fig. 8E) and dabrafenib treatment led to a drastic reduction in their absolute numbers (Fig. 8F). Like the bone marrow, the majority of circulating MDSCs were Ly6C^int^Ly6G^hi^ PMN lineage (83.9%, SD ± 6.6) while Ly6C^hi^Ly6G^neg^ m-MDSCs were a relatively minor fraction of the MDSC pool (4.0%, SD ± 1.9; Fig. 8G and H). Dabrafenib treatment selectively decreased PMN-MDSC percentages in the blood (Fig. 8H); which was reflected in the significant drop in absolute numbers of PMN-MDSCs per µL of blood (Fig. 8I). In the tumor microenvironment, dabrafenib treatment reduced MDSC numbers by 50% (Fig. 8J). Like the blood, PMN-MDSCs were the majority MDSC population; however, there was an enrichment for m-MDSCs which were present at numbers comparable to PMN-MDSCs (∼3,897 cell/mg tumor and 5,455 cell/mg tumor for PMN- and m-MDSCs, respectively). In tumors from dabrafenib-exposed mice, the percentage of m-MDSCs increased with a corresponding decrease in PMN-MDSCs (Fig. 8K). Absolute numbers of PMN-MDSCs per mg of tumor were reduced 5-fold by dabrafenib compared with controls while m-MDSCs showed a nonsignificant reduction in dabrafenib-treated animals (Fig. 8L)

We also tested dabrafenib in the YUMMER melanoma model. YUMMER cells are derived from YUMM1.7 cells exposed to UVB radiation resulting in 1,446 unique nonsynonymous exonic mutations (47). This model is more inflammatory compared with YUMM1.7 tumors with greater immune infiltration. In contrast to YUMM1.7 tumors, the majority of YUMMER tumor MDSCs were m-MDSC with very few Ly6G^+^ PMN-MDSC (Fig. 8M). Treatment with dabrafenib caused a significant decrease in m-MDSCs, which showed increased Ly6G expression (Fig. 8M). There was also the emergence of a Ly6G^+^Ly6C^neg^ population when mice received dabrafenib (Fig. 8M) similar to the *in vitro* data (Fig. 1K) showing dabrafenib was driving the development of a more mature PMN population in the intratumoral MDSC population. Thus, cumulatively the data in the YUMM1.7 and YUMMER models shows that dabrafenib impacts MDSC accumulation in the tumor microenvironment *in vivo* with a significant impact on PMN populations.

## Discussion

In this article, we describe an off-target effect of dabrafenib that impacts MDSC development by activation of the stress kinase GCN2, revealing developmental and transcriptional lineage relationships relevant for differentiation and function. Dabrafenib-induced GCN2 activity reduced ribosome association with transcripts and altered transcriptional programs preventing developmental transition from immature myeloid progenitors resulting in loss of PMN-MDSC differentiation and MDSC suppressive activity. Our findings are consistent with data from other groups showing cancer drives expansion of PMN-MDSCs from monocytic precursors, although we did not find that monocytic precursors expressed c-kit (CD117), suggesting in our model the monocyte PMN-MDSC precursors exist in a more mature state than the monocyte-like progenitors described by Mastio and colleagues (45). Nevertheless, our data clearly show an intimate relationship between PMN- and m-MDSCs, and highlights the functional balance between GCN2 activity, ISR signaling, and maturation of MDSC populations.

ISR signaling is critical for cells to balance demands of metabolic activity versus limitations of nutrient availability, protein translation, and mitochondrial stress (48). We have previously shown that loss of GCN2 in mature monocytic and PMN lineages alters MDSC transcriptional profiles with attenuation of immune suppressive function and increased expression of inflammatory cytokines (5). This effect was indirect, resulting from transcriptional alteration of metabolic transcriptional programming reducing oxidative respiration (5). In the current article, we found that dabrafenib significantly increased general oxidative metabolism in MDSCs with a specific increase in oxidative phosphorylation transcriptional signatures in the same immature cell populations exhibiting inhibited developmental progression. This suggests that precise metabolic regulation is required to maintain inflammatory differentiation capacity, and dabrafenib-GCN2–driven hyperactivation of oxidative metabolism dysregulates inflammation-driven myelopoiesis.

The transcription regulator Rb protein restricts PMN-MDSC development, controlling relative composition of monocytic versus PMN populations. In tumor-bearing conditions, *Rb1* epigenetic silencing favors PMN-MDSC development (49) and its deletion promotes accumulation of monocytic precursors that can give rise to PMN-MDSCs (45). We observed a significant reduction of the *Rb1* regulon in a key intermediate PMN cluster (cluster 9) after dabrafenib therapy. However, the *Rb1* regulon was not identified in the cluster 6 to cluster 9 trajectory branch suggesting differential Rb activity was not driving PMN population maturation. Moreover, cluster 9 showed the most significant dabrafenib-mediated loss of regulon activity indicating that transcriptional programming is highly impacted in this cell population.

There were relatively few regulons increased by dabrafenib with the most prominent being regulons that would be predicted to impact metabolism and cell identity (*Atf5*, *Mafg*, and *Zbtb7a*). In contrast, dabrafenib downregulated a number of regulons associated with *Jun* and *Fos* transcription factors, redox responses, metabolism, and cellular differentiation. While we observed a general reduction of ribosome association with mRNA, we did not observe a loss of relative polysome assembly, suggesting a decrease in translation but no specific inhibition of ribosome assembly. Thus, alterations observed are likely due to GCN2-induced transcriptional programs. The *Atf5* regulon was the only regulon increased in all dabrafenib-treated cell clusters suggesting strong, generalized induction. ATF5 is a key driver of the ISR transcriptional response, impacting mTOR function and oxidative metabolism in mammalian cells (50). Importantly, ATF5 also promotes cell survival, and its downregulation is required for astrocyte differentiation from neural progenitors (51). In addition, the amino acid starvation response is enriched in hematopoietic stem cells, promoting stem cell survival, with a rapid diminution as stem cells differentiate to common myeloid progenitors and finally to mature monocytic and PMN populations (52). This correlated with reduced GCN2 expression in stem cells versus progenitor populations suggesting genetic control of GCN2 and eIF2α phosphorylation is directly associated with differentiation (52). Thus, dysregulation of GCN2 and ATF5 activity would be expected to impact differentiation programs suggesting that this feature of dabrafenib may have prominent effects on MDSC differentiation.

Congenital loss of GCN2 does not impact basal immune cell composition in the periphery, and here we show GCN2 deficiency does not affect MDSC composition ± dabrafenib. This contrasts with the ISR kinase PERK, which is required for tumor-driven myelopoiesis and MDSC development in the spleen of mice (53). Complementation studies have suggested that GCN2 and PERK serve compensatory roles in sensing cellular stress (54). Indeed, Reactome analysis of MDSCs suggested dabrafenib induced a UPR and PERK transcriptional signature suggesting increased PERK activity; however, we previously reported that in MDSCs PERK does not drive compensatory nutrient starvation responses in the absence of GCN2 function, and likewise loss of GCN2 has no impact on UPR responses in the presence or absence of PERK (5). This agrees with the data presented here showing that loss of GCN2 abrogated the effect of dabrafenib on ISR signaling and MDSC development. Thus, taken as a whole, the data suggest that the PERK and GCN2 branches of the ISR have distinct biologic roles in MDSC development and function. Our study shows a loss of suppressive activity when MDSCs develop in the presence of dabrafenib resulting from proliferative arrest and the loss of functionally competent MDSC populations. Likewise in this article, we show that when dabrafenib is added to mature MDSCs there is an effect on their ability to suppress T-cell responses. Coupled with the lack of impact on MDSC development, the data suggest that GCN2 function in the tissue microenvironment is an important factor regulating phenotype, but increased activity during development disrupts transcriptional and metabolic programs leading to attenuated development. The development of highly specific GCN2 agonists will bring more clarity to thresholds of activation necessary in each context.

In this article, we took advantage of off-target effects of dabrafenib to probe MDSC developmental relationships and function related to GCN2 activity. The data reveal novel insights regarding the relative balance of ISR signaling and MDSC development that may have therapeutic relevance. Dabrafenib therapy in BRAF-mut melanoma increases T-cell infiltration and sensitizes the tumors to immune checkpoint inhibition therapy (55, 56). However, dabrafenib does not affect human T-cell function *in vitro* suggesting alternative tumor-intrinsic or off-target effects are impacting antitumor immunity (57). Our data suggest immune-stimulating effects of dabrafenib therapy may be at least partially due to altered MDSC function, a prediction supported by the reduction of PMN-MDSCs in BRAF-mut tumor-bearing mice after dabrafenib treatment. In addition, we have shown GMCSF dependency for dabrafenib-mediated GCN2 activation effects on MDSC differentiation. Therefore, it is plausible that other myeloid cells expanded under such cytokine are also conditioned by such hyperactivation leading to a dysregulated phenotype. Human DCs are not affected by dabrafenib *in vitro* (57). In tumor-associated macrophages, GCN2 is critical to regulate immune response in the tumor microenvironment (5). It can be theorized that, similar to observations in MDSCs, dabrafenib-mediated GCN2 hyperactivation could induce more immature states of differentiation that might contribute to better antitumoral response. Ultimately, a deeper understanding regarding how tumor–stroma interactions are impacted by dabrafenib in specific and off-target mechanisms will improve clinical management of the disease. Thus, further studies will be key to determining whether other kinase inhibitors showing GCN2 off-target activation result in similar beneficial repercussions on the immune response.

## Supplementary Material

Supplementary Figure 1 Supplementary Figure 1

Supplementary Figure 2 Supplementary Figure 2

Supplementary Figure 3 Supplementary Figure 3

Supplementary Figure 4 Supplementary Figure 4

Supplementary Table s1 Supplementary Table s1

Supplementary Table s2 Supplementary Table s2

Supplementary Table s3 Supplementary Table s3

Supplementary Table s4 Supplementary Table s4
