# Supplementary material for: Dabrafenib Alters MDSC Differentiation and Function by Activation of GCN2
Source: Cancer Res Commun. 2024 Mar 13;4(3):765–84. doi: 10.1158/2767-9764.CRC-23-0376 (PMC10936428; doi:10.1158/2767-9764.CRC-23-0376)
Supplement: Supplementary Figure 1 [file crc-23-0376-s01.pdf]

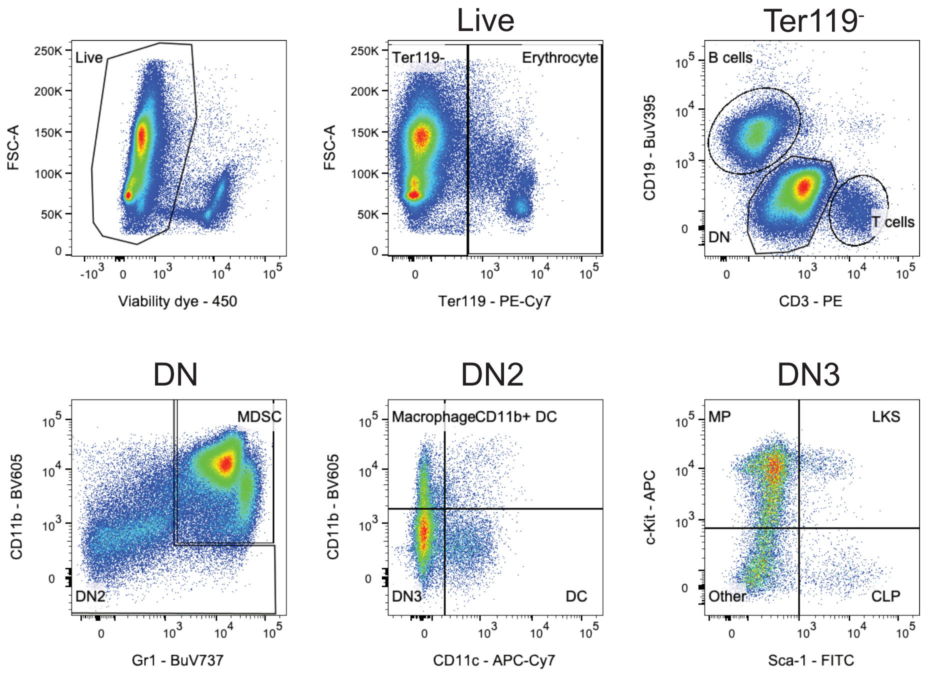

Supplementary Figure s1. Gating strategy used to analyze immune cell subpopulation in fresh bone marrow and MDSCs in vitro cultures.
