# Supplementary material for: Dabrafenib Alters MDSC Differentiation and Function by Activation of GCN2
Source: Cancer Res Commun. 2024 Mar 13;4(3):765–84. doi: 10.1158/2767-9764.CRC-23-0376 (PMC10936428; doi:10.1158/2767-9764.CRC-23-0376)
Supplement: Supplementary Figure 2 [file crc-23-0376-s02.pdf]

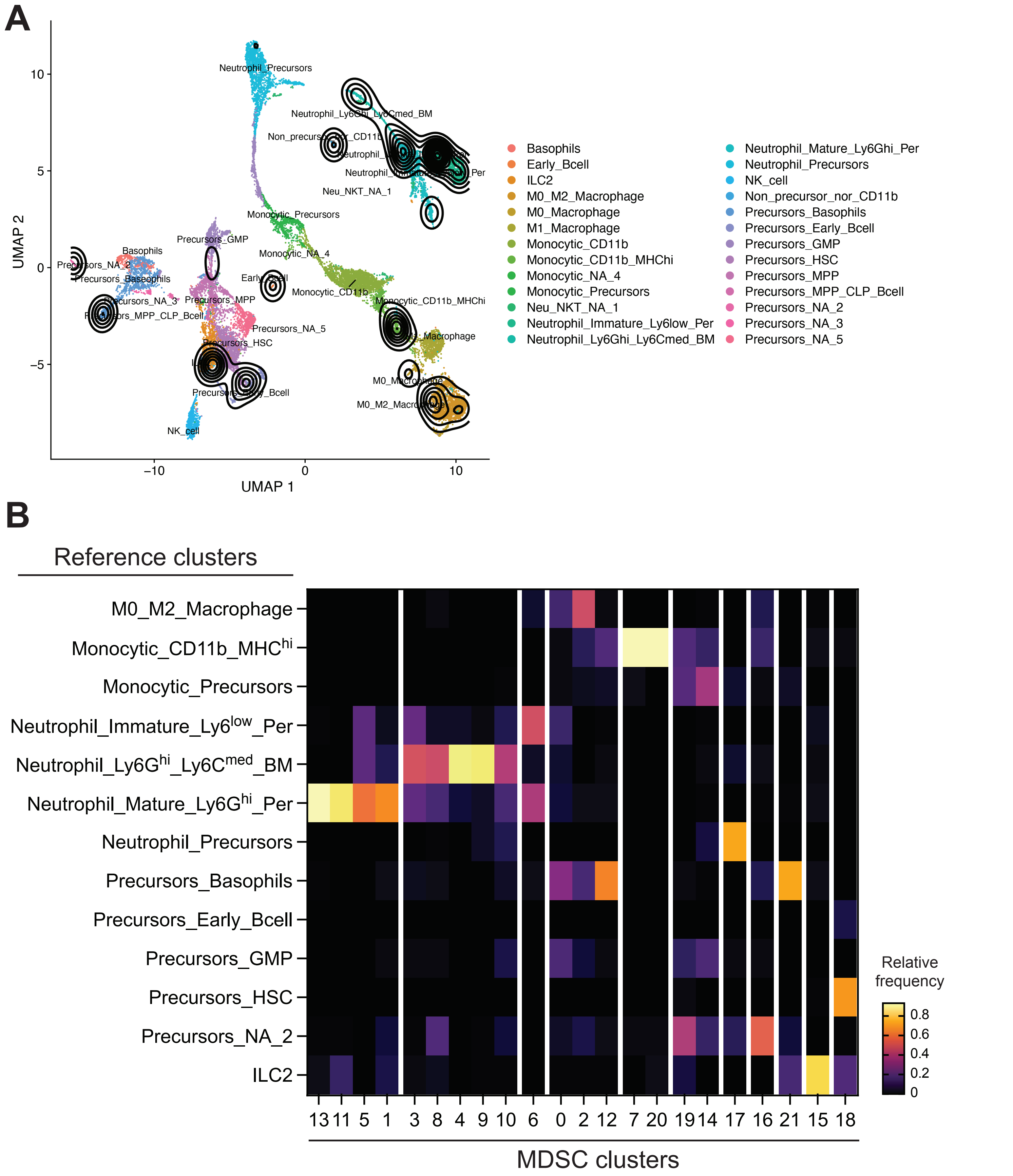

Supplementary Figure s2. Cluster annotation strategy. (A) UMAP plot representing the FastMNN-integrated scRNA data built from publicly available data sets (Supplemental Table D). The 26 resulting clusters were annotated based on the markers used in sorting strategy and the differential expressed genes among clusters. Integrated MDSC scRNAseq data (contour lines) was projected onto the integrated reference data set using FindTransferAnchors. (B) Heatmap of the frequency of cells within each MDSC cluster that is projected onto the reference annotated groups. Scale represents relative frequency.
