# Supplementary material for: Dabrafenib Alters MDSC Differentiation and Function by Activation of GCN2
Source: Cancer Res Commun. 2024 Mar 13;4(3):765–84. doi: 10.1158/2767-9764.CRC-23-0376 (PMC10936428; doi:10.1158/2767-9764.CRC-23-0376)
Supplement: Supplementary Figure 3 [file crc-23-0376-s03.pdf]

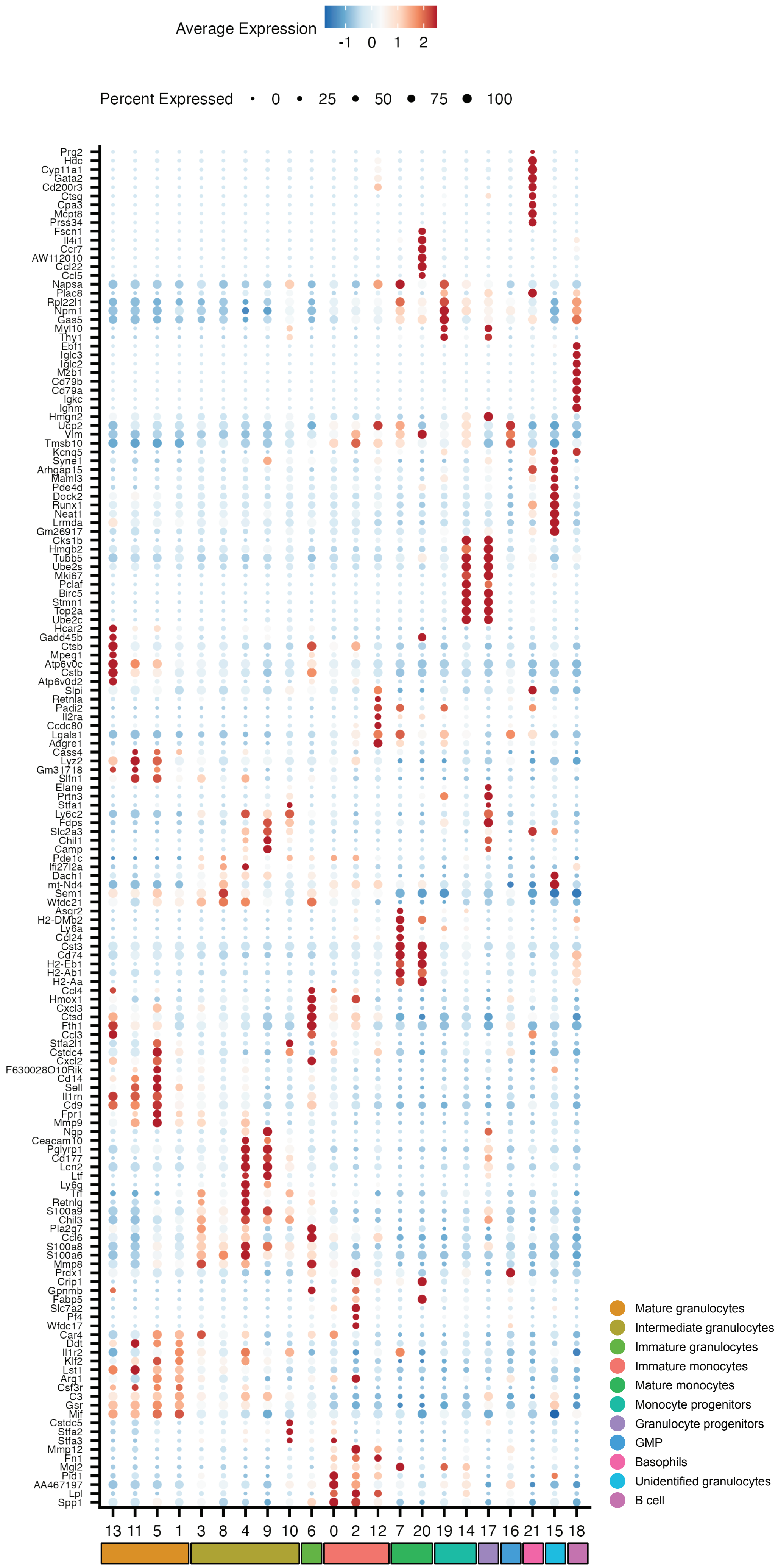

Supplementary Figure s3. Top marker genes used for cluster identification. Normalized expression and per cluster percentage expression of top 10 marker genes for each cell cluster for the scRNA sequencing analysis on the integrated sample including MDSCs generated ± 1.5μM DAB.
