# Supplementary material for: Dabrafenib Alters MDSC Differentiation and Function by Activation of GCN2
Source: Cancer Res Commun. 2024 Mar 13;4(3):765–84. doi: 10.1158/2767-9764.CRC-23-0376 (PMC10936428; doi:10.1158/2767-9764.CRC-23-0376)
Supplement: Supplementary Figure 4 [file crc-23-0376-s04.pdf]

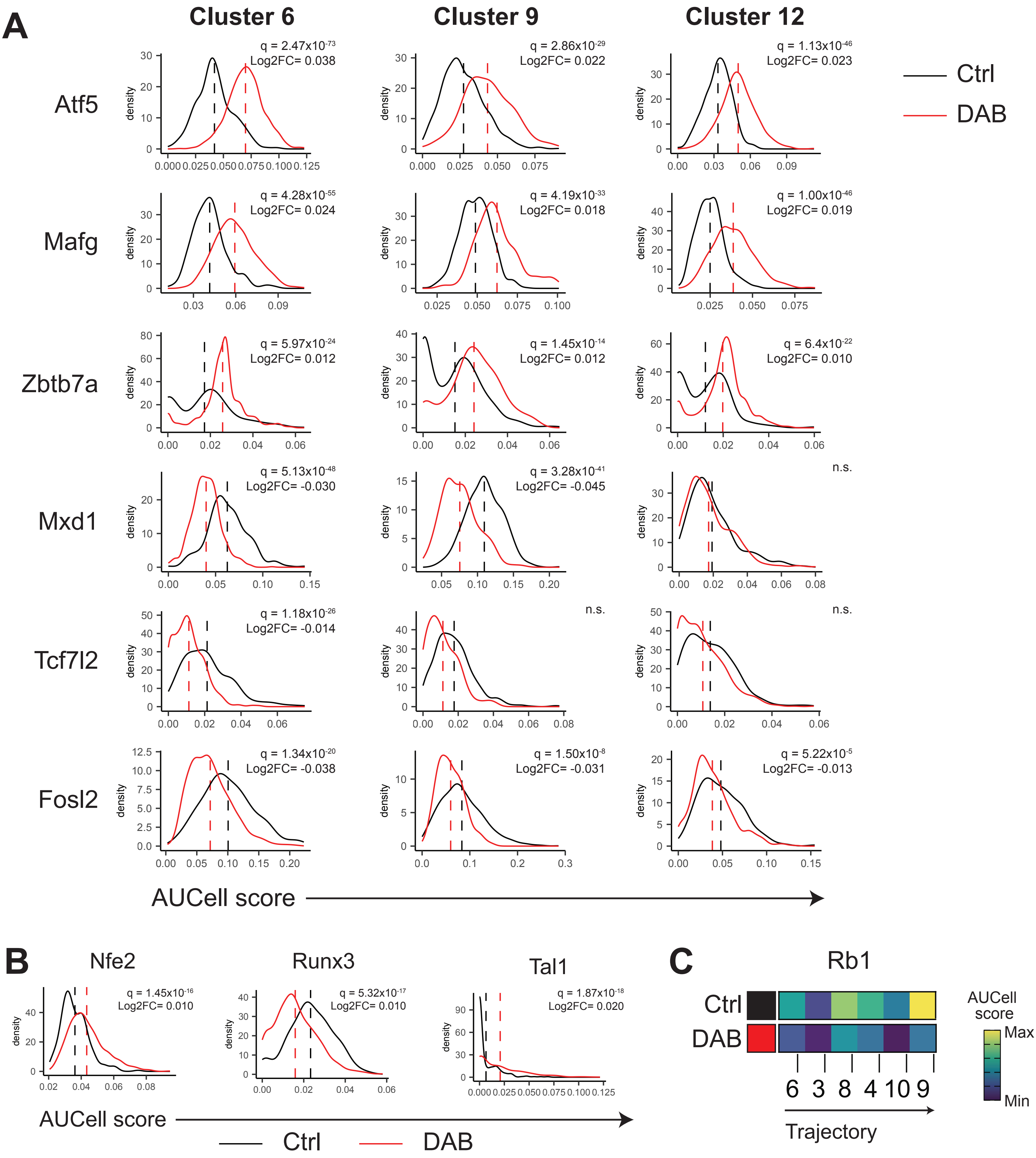

Supplementary Figure s4. Selected regulon AUCell enrichment scores of cells in Ctrl or dabrafenib (DAB) conditions. (A) Density plots representing the distribution of the AUCell score for dabrafenib-induced enriched regulons (Atf5, Mafg and Zbtb7a) and Ctrl-enriched regulons (Mxd1, Tcf7l2 and Fosl2) in representative PMN (cluster 6 and 9) and monocyte clusters (cluster 12). Dashed line indicates geometric mean for Ctrl (black) or DAB (red). Exact q value and average Log2FC is indicated in each panel. No significance is indicated as “n.s”. Statistics show Wilcoxon test. (B) Density plots representing the distribution of the AUCell score for dabrafenib-modulated regulons in monocytic cluster 12 (Nfe2, Runx3 and Tal1). Dashed line indicates geometric mean for Ctrl (black) or DAB (red). Exact q value and average Log2FC is indicated in each panel. Statistics show Wilcoxon test. (C) Heatmap of the Rb1 regulon during differentiation in the direction from node a to node b in Figure 6C, which spans from cluster 6 (immature granulocytes) to cluster 9 (intermediate granulocytes) in control cell. Scale represents z-score of the normalized average AUCell score per regulon.
